# Dual Structure of a Vanadyl-Based Molecular Qubit Containing a Bis(β-diketonato) Ligand

**DOI:** 10.1021/acs.inorgchem.4c00834

**Published:** 2024-04-15

**Authors:** Manuel Imperato, Alessio Nicolini, Matteo Boniburini, Daniele Sartini, Enrico Benassi, Mario Chiesa, Lara Gigli, Yu-Kai Liao, Arsen Raza, Enrico Salvadori, Lorenzo Sorace, Andrea Cornia

**Affiliations:** † Dipartimento di Scienze Chimiche e Geologiche e UdR INSTM, Università degli Studi di Modena e Reggio Emilia, via G. Campi 103, 41125 Modena, Italy; ‡ Dipartimento di Scienze Fisiche, Informatiche e Matematiche, Università degli Studi di Modena e Reggio Emilia, via G. Campi 213/A, 41125 Modena, Italy; § Dipartimento di Chimica “Ugo Schiff” e UdR INSTM, Università degli Studi di Firenze, via della Lastruccia 3, 50019 Sesto Fiorentino (FI), Italy; ∥ Dipartimento di Chimica e NIS Centre, Università degli Studi di Torino, via P. Giuria 7, 10125 Torino, Italy; ⊥ Elettra-Sincrotrone Trieste S.C.p.A., Strada Statale 14 - km 163.5 in AREA Science Park, 34149 Basovizza (TS), Italy

## Abstract

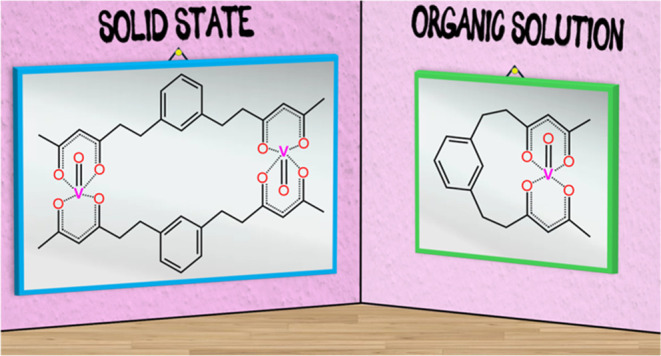

We designed [VO(bdhb)] (**1**′) as a new electronic qubit containing an oxovanadium(IV) ion (*S* = 1/2) embraced by a single bis(β-diketonato) ligand [H_2_bdhb = 1,3-bis(3,5-dioxo-1-hexyl)benzene]. The synthesis afforded three different crystal phases, all of which unexpectedly contain dimers with formula [(VO)_2_(bdhb)_2_] (**1**). A trigonal form (**1*****h***) with a honeycomb structure and 46% of solvent-accessible voids quantitatively transforms over time into a monoclinic solvatomorph **1*****m*** and minor amounts of a triclinic solventless phase (**1*****a***). In a static magnetic field, **1*****h*** and **1*****m*** have detectably slow magnetic relaxation at low temperatures through quantum tunneling and Raman mechanisms. Angle-resolved electron paramagnetic resonance (EPR) spectra on single crystals revealed signatures of low-dimensional magnetic behavior, which is solvatomorph-dependent, being the closest interdimer V···V separations (6.7–7.5 Å) much shorter than intramolecular V···V distances (11.9–12.1 Å). According to ^1^H diffusion ordered spectroscopy (DOSY) and EPR experiments, the complex adopts the desired monomeric structure in organic solution and its geometry was inferred from density functional theory (DFT) calculations. Spin relaxation measurements in a frozen toluene-*d*_8_/CD_2_Cl_2_ matrix yielded *T*_m_ values reaching 13 μs at 10 K, and coherent spin manipulations were demonstrated by Rabi nutation experiments at 70 K. The neutral quasi-macrocyclic structure, featuring nuclear spin-free donors and additional possibilities for chemical functionalization, makes **1**′ a new convenient spin-coherent building block in quantum technologies.

## Abstract 2

**Designed to perform as a qubit**, the vanadyl complex of a flexible bis-chelating ligand displays quantum spin coherence in organic solution, with phase memory time >10 μs at 10 K and Rabi oscillations visible in nutation experiments at 70 K. Upon crystallization, molecules unexpectedly rearrange into dimers with a large V···V separation (∼1.2 nm). The crystalline material behaves as an extended magnetic solid due to the short intermolecular V···V contacts but retains slow spin−lattice relaxation.

## Introduction

The properties of simple paramagnetic d-block metal complexes have witnessed a renaissance of interest since the discovery that two- or multilevel molecular spin systems are potential platforms for quantum technologies.^1−6^ The simplest realization of a two-level system (*qubit*) is an *S* = 1/2 metal center such as vanadium(IV) or copper(II). Vanadyl (VO^2+^) complexes exhibit particularly good quantum coherence properties,^7−9^ with phase memory times (*T*_m_) that can exceed 100 μs, although only in special, nuclear spin-free environments.^10^

**Chart 1:**
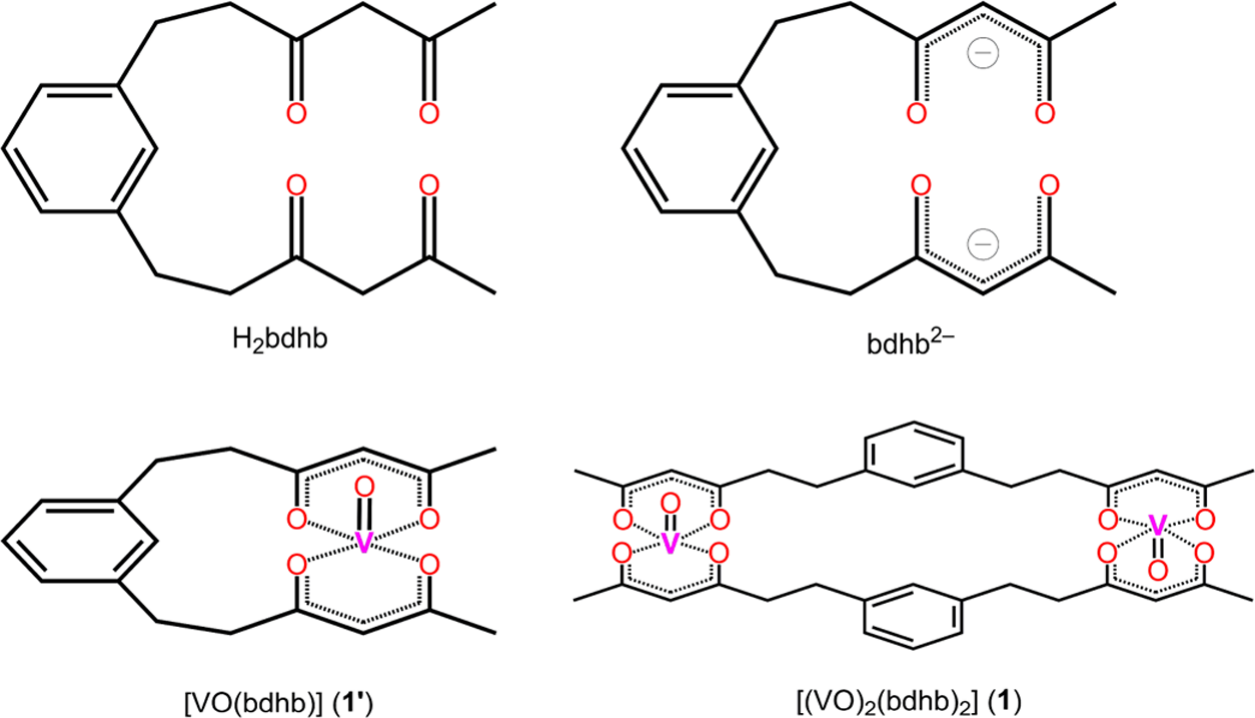
Perspective Structures of the H_2_bdhb Proligand, the bdhb^2–^ Ligand Obtained by Double Deprotonation, and the Monomeric (**1**′) and Dimeric (**1**) 1:1 Adducts between bdhb^2–^ and VO^2+^

In our search for new molecular architectures displaying quantum spin coherence, we have studied the coordination chemistry of a bis(β-diketonato) ligand (bdhb^2–^) obtained by double deprotonation of tetraketone H_2_bdhb (Chart 1). This organic molecule was first synthesized in 1977 by Alberts and Cram^11,12^ and contains two Hacac-like moieties linked to a 1,3-phenylene unit (Hacac = acetylacetone). Previous synthetic and solution studies demonstrated that H_2_bdhb interacts with several metal ions, including those of the d- and f-blocks.^11,13−15^ As a consequence of the two preorganized and covalently linked β-diketonato units, these complexes have larger formation constants as compared with simple acetylacetonates. From the perspective of quantum technologies, they are also attractive because they have a neutral charge, nuclear spin-free donors, and additional possibilities for chemical functionalization on the aromatic ring. However, detailed structural information is extremely scarce and, to the best of our knowledge, no crystal structures have been reported.

We herein show that bdhb^2–^ and VO^2+^ yield a 1:1 adduct whose structure is phase-dependent. In the crystalline state, the compound exists as [(VO)_2_(bdhb)_2_] (**1**) dimers (Chart 1) containing widely separated metal ions (ca. 12 Å), as shown by the X-ray analysis of three different crystal phases, including a nanoporous solvatomorph. The crystalline material displays slow magnetic relaxation in a static magnetic field and low-dimensional magnetic behavior attributed to short intermolecular V···V contacts. However, in organic solution, it exists as monomeric species [VO(bdhb)] (**1**′), as independently demonstrated by ^1^H diffusion ordered spectroscopy (DOSY) and continuous-wave (CW) electron paramagnetic resonance (EPR) spectroscopy (Chart 1). In a frozen toluene-*d*_8_/CD_2_Cl_2_ matrix at 10 K, **1**′ exhibits relaxation times *T*_1_ = 14 ms and *T*_m_ = 13 μs, as measured by pulsed EPR spectroscopy, and shows quantum spin coherence up to 70 K as demonstrated by Rabi nutations.

## Experimental Section

### General Procedures

All chemicals were of reagent grade and used as received, unless otherwise noted. CH_2_Cl_2_ was purchased anhydrous, while Et_2_O was dried using standard methods.^16^ CD_2_Cl_2_ (99.8%D) and toluene-*d*_8_ (99.5%D) were used as received. CDCl_3_ (99.8%D) was passed through a column of basic alumina prior to use.^16^ CH_2_Cl_2_, Et_2_O, and deuterated solvents were deoxygenated through three freeze–pump–thaw cycles and stored over activated 4 Å molecular sieves. The amount of water in commercial VOSO_4_·*x*H_2_O was determined by combustion analysis (*x* = 3.6). Crystallizations were carried out inside a dinitrogen-filled MBraun UNILab glovebox. 1,3-Bis(3,5-dioxo-1-hexyl)benzene (H_2_bdhb) was prepared by condensation of 1,3-bis(bromomethyl)benzene with Hacac, as described by Alberts and Cram.^11,12^ The crude material was purified by gradient flash chromatography (silica gel, CH_2_Cl_2_/acetone) rather than by the lengthy, four-step procedure reported by these authors (72% yield). Combustion analysis was performed using a Thermo Fisher Scientific Flash 2000 analyzer. Fourier transform infrared (FT-IR) spectra were collected in attenuated total reflectance (ATR) mode on a JASCO 4700 FT-IR spectrometer, between 400 and 4000 cm^–1^ and with a resolution of 2 cm^–1^. Electronic spectra were collected in dry CH_2_Cl_2_ using a Jasco V-570 ultraviolet–visible–near–infrared (UV–vis–NIR) spectrometer operating in double-beam mode (optical path length *l* = 0.1 cm). ^1^H NMR spectroscopy measurements were conducted in CD_2_Cl_2_ or CDCl_3_ at 298 K on an AVANCE400 FT-NMR spectrometer from Bruker Biospin (400.13 MHz), using 5 mm airtight Young-valved NMR tubes from Norell, to prevent contact with moisture and/or dioxygen. Spectra were analyzed using TopSpin (version 4.3.0).^17^ The chemical shifts (δ) are expressed in ppm downfield from tetramethylsilane (TMS) as external standard, setting the residual ^1^H signals of CD_2_Cl_2_ and CDCl_3_ at 5.32 and 7.26 ppm, respectively.^18^ Alternatively, TMS was added as an internal standard. ^1^H DOSY measurements were carried out at 400.13 MHz and 298 K with a *ledbpgp2s* sequence (Bruker library) using bipolar gradient pulses.^19^ The diffusion time (big delta = 0.010 and 0.060 s for **1*****h*** and H_2_bdhb, respectively) and the gradient length (small delta = 2000 and 1000 μs for **1*****h*** and H_2_bdhb, respectively) were tuned through the monodimensional sequence *ledbpgp2s1d* (Bruker library). The signal decay was fitted with a single exponential function using Bruker Dynamic Center software (version 2.8.3). Diffusion coefficients were normalized using TMS as an internal reference, following the procedure described by Stalke et al.^20^ The following abbreviations are used in reporting spectroscopic data: br = broad, sh = shoulder. In addition, specific abbreviations are used for ^1^H NMR (s = singlet, t = triplet, q = quartet) and FT-IR data (s = strong, m = medium, w = weak).

### Synthesis of VO(bdhb)·0.35EtOH·0.35H_2_O

In a 10 mL round-bottom flask equipped with a magnetic stirrer, VOSO_4_·3.6H_2_O (70.0 mg, 0.307 mmol) was dissolved in H_2_O (1.5 mL) to give a light blue solution which was heated to 80 °C. NaOAc·3H_2_O (63.0 mg, 0.463 mmol) was added to this solution, which turned dark blue/gray and had pH = 4. H_2_bdhb (60.7 mg, 0.201 mmol) was separately added to a H_2_O/EtOH mixture (1:1 v/v, 2 mL) to yield an orange emulsion. This emulsion was then added dropwise to the vanadyl solution, causing the immediate precipitation of a sticky green solid. The obtained suspension was stirred at 80 °C for 4 h and then allowed to cool down to room temperature (RT). The dark green precipitate was collected by filtration through a fritted glass funnel (porosity G4), washed with H_2_O (10 mL), and dried under vacuum to give a green powder (57.7 mg, 74%). Anal. Calcd for VO(bdhb)·0.35EtOH·0.35H_2_O: C, 57.63; H, 5.90. Found: C, 57.59; H, 5.88%. FT-IR (ATR) ν̃_max_ (cm^–1^): 3455 (m, br), 2957 (w), 2925 (w), 2858 (w), 1705 (w), 1553 (s), 1515 (s), 1434 (sh), 1383 (s), 1360 (s), 1339 (sh), 1290 (m), 1259 (m), 1173 (w), 1158 (w), 1145 (w), 1090 (w), 1022 (m), 998 (s), 976 (m), 955 (m), 901 (m), 884 (m), 788 (m), 717 (w), 703 (m), 668 (m), 662 (m), 610 (m), 480 (s), 473 (s), 458 (s), 452 (s), 442 (m), 432 (m), 427 (m), 422 (m), 416 (m), 409 (m), 402 (m). UV–vis–NIR (CH_2_Cl_2_, 2.76 × 10^–4^ M) λ_max_, nm (ε, M^–1^cm^–1^): 304 (2.72 × 10^4^). UV–vis–NIR (CH_2_Cl_2_, 2.76 × 10^–3^ M) λ_max_, nm (ε, M^–1^cm^–1^): ∼420 (sh, ∼175), 580 (53.9), 683 (44.8).

### Synthesis of [(VO)_2_(bdhb)_2_]·1.90Et_2_O·0.13CH_2_Cl_2_ (**1*****h***)

Inside the glovebox, VO(bdhb)·0.35EtOH·0.35H_2_O (188.3 mg, 0.4832 mmol) was dissolved in dry CH_2_Cl_2_ (10.5 mL) to give a dark green solution, which was then stirred for 30 min and filtered. Upon slow diffusion of dry Et_2_O vapors, large X-ray-quality light blue rods of **1*****h*** formed after approximately 2 weeks. Crystals were separated, dried under a dinitrogen stream, and stored in a sealed vial at −80 °C (77.9 mg, 36%). Anal. Calcd for **1**·1.90Et_2_O·0.13CH_2_Cl_2_: C, 59.25; H, 6.74. Found: C, 59.06; H, 6.79%. The amount of crystallization solvent was confirmed also by ^1^H NMR spectroscopy (Figure S2). FT-IR (ATR) ν̃_max_ (cm^–1^): 2971 (w), 2924 (w), 2857 (w), 1553 (s), 1517 (s), 1423 (sh), 1386 (s), 1360 (s), 1339 (s), 1297 (m), 1276 (m), 1261 (m), 1181 (w), 1141 (w), 1115 (w), 1090 (w), 1025 (m), 995 (s), 956 (m), 944 (m), 901 (m), 883 (m), 806 (m), 795 (m), 764 (m), 750 (m), 719 (m), 704 (m), 667 (m), 621 (m), 498 (s), 483 (s), 472 (s), 450 (s), 445 (m), 441 (m), 433 (m), 426 (m), 420 (m), 414 (m), 405 (w). UV–vis–NIR (CH_2_Cl_2_, 2.80 × 10^–4^ M) λ_max_, nm (ε, M^–1^cm^–1^): 304 (2.96 × 10^4^). UV–vis–NIR (CH_2_Cl_2_, 2.80 × 10^–3^ M) λ_max_, nm (ε, M^–1^cm^–1^): 396 (93.8), 593 (42.0), 683 (44.6). ^1^H NMR (400.13 MHz, CDCl_3_, 298 K) δ (ppm): 7.1 (br, Ar–H), 6.7 (br, Ar–H), 5.29 (s, 2H, CH_2_Cl_2_), 3.47 (q, 4H, CH_2_, Et_2_O), 3.3 (br), 2.8 (br), 2.3 (br, CH_3_), 1.20 (t, 6H, CH_3_, Et_2_O). ^1^H NMR (400.13 MHz, CD_2_Cl_2_, 298 K) δ (ppm): 7.2 (br, Ar–H), 6.8 (br, Ar–H), 5.33 (s, 2H, CH_2_Cl_2_), 3.43 (q, 4H, CH_2_, Et_2_O), 3.0 (br), 2.5 (br), 2.3 (br, CH_3_), 1.15 (t, 6H, CH_3_, Et_2_O).

Upon prolonged standing in their mother solution at RT, the crystals of **1*****h*** were found to convert into blue-green parallelogram-shaped crystals of a monoclinic solvatomorph (**1*****m***). Anal. Calcd for **1**·0.80Et_2_O·0.20CH_2_Cl_2_: C, 58.36; H, 6.02. Found: C, 58.34; H, 5.86%. The amount of crystallization solvent was confirmed also by ^1^H NMR spectroscopy (Figure S3). ^1^H NMR (400.13 MHz, CDCl_3_, 298 K) δ (ppm): 7.1 (br, Ar–H), 6.7 (br, Ar–H), 5.30 (s, 2H, CH_2_Cl_2_), 3.47 (q, 4H, CH_2_, Et_2_O), 3.3 (br), 2.8 (br), 2.3 (br, CH_3_), 1.21 (t, 6H, CH_3_, Et_2_O).

### X-ray Crystallography

Powder X-ray diffraction (PXRD) data were collected at RT on a Bruker D8 DAVINCI instrument with Cu–Kα radiation, working in a θ–θ configuration. Polycrystalline powders were obtained by grinding crystals collected from the mother solution. The quantitative phase analysis (QPA) of the powder pattern (Figure S12) was performed using the GSAS II software.^21^ Both the **1*****m*** and solventless **1*****a*** forms were used as starting structural models. The **1*****h*** form was initially inserted in the refinement, but after some cycles its fraction turned out to be close to zero; for this reason, it was excluded in the final refinement. The GSAS refinement was carried out according to the following features: (i) the background was fitted with a Chebyshev function with 26 coefficients; (ii) the peak profiles were modeled using a pseudo-Voigt function with one Gaussian and one Lorentzian component; (iii) the lattice parameters, the phase fractions, and the coefficient corresponding to sample displacement were also refined. Single-crystal X-ray diffraction data were collected on a Bruker D8 VENTURE instrument with Cu–Kα radiation (λ = 1.54178 Å) at 100 K (Table S1). Crystals were soaked in NVH immersion oil (Jena Bioscience) and mounted on a polyethylene ring. Structure solution and full matrix least-squares refinement on *F*_o_^2^ were based on standard methods using SHELXT-2018/2,^22^ SHELXL-2018/3,^23^ and the WINGX v2020.2 suite.^24^ Most nonhydrogen atoms were refined anisotropically, while hydrogen atoms were added in geometrically idealized positions and treated as riding contributors with isotropic displacement parameter (IDP) tied to 1.5 or 1.2 times the *U*_eq_ value of the parent atom for CH_3_ and other hydrogens, respectively. A rotating group refinement (AFIX 137) was adopted for CH_3_ groups of bdhb^2–^ ligands, unless otherwise noted. Specific refinement details for structures **1*****h***, **1*****m***, and **1*****a*** are reported hereafter. Intermolecular contacts were calculated using PARST.^25^ Molecular structures and packing diagrams were drawn using ORTEP-3 for Windows v2023.1.^24^

### **1*****h***

Complete refinement of the ordered part of the structure left several electron density peaks of up to 4.6 *e*Å^–3^ arising from disordered solvent that fills the channel-like voids. These voids amount to 5932 Å^3^ per unit cell, or 46% of the unit cell volume (grid size = 0.20 Å, probe radius = 1.20 Å). The indices were by consequence very high, with *w*R**2 = 0.4261 (all data) and *R*1 = 0.1212 (*I* > 2σ(*I*)). Attempts to develop an atomistic model for the solvent were unsuccessful and the contribution from disordered solvent was handled using the SQUEEZE^26^ procedure implemented in PLATON,^27^ which lowered the indices dramatically to *w*R**2 = 0.0979 (all data) and *R*1 = 0.0311 (*I* > 2σ(*I*)). The SQUEEZE procedure gave 1099 electrons per unit cell, which correspond to ca. 26 solvent molecules, or ca. 2.9 solvent molecules per dimeric unit (Et_2_O and CH_2_Cl_2_ both have 42 electrons). Combined use of elemental analysis and ^1^H NMR spectroscopy provided 1.90 Et_2_O and 0.13 CH_2_Cl_2_ molecules per dimer, i.e., a 30% lower solvent content. It is well possible that a fraction of solvent in the channels was quickly lost as the samples used for elemental analysis and ^1^H NMR were dried. By contrast, the individual selected for X-ray diffraction was removed from the mother liquid, immediately soaked in immersion oil, and transferred to the cold dinitrogen stream of the diffractometer.

### **1*****m***

The lattice Et_2_O and CH_2_Cl_2_ molecules are disordered around an inversion center so that the sum of their site occupancy factors (SOFs) cannot exceed 0.5. Preliminary independent refinement of their SOFs indicated maximal occupancy of this crystallographic site; the two SOFs were then forced to sum up to 0.5 and converged to 0.447(5) and 0.053(5), respectively. Restraints were applied to the geometry of these solvent molecules based on the structure in CCDC 973959,^28^ and to the anisotropic displacement parameters (ADPs) of C and O atoms in Et_2_O, using RIGU instruction.^29^ The chlorine atoms of CH_2_Cl_2_ were refined isotropically and with the same IDP. The positions of an Et_2_O methyl carbon and of CH_2_Cl_2_ carbon atom were found to virtually coincide; to ensure convergence, the two atoms were constrained to have the same coordinates and the same ADPs.

### **1*****a***

One of the two independent methyl groups was found disordered over two positions rotated from each other by 60° and was refined using AFIX 127 instruction with best-fit occupancies 0.61(4):0.39(4).

### Magnetic Measurements

Direct current (dc) magnetic data were collected on a crushed crystalline sample of **1*****h*** (5.51 mg) wrapped in poly(tetrafluoroethylene) (PTFE) tape by using a QD MPMS magnetometer. Raw data were corrected for the diamagnetic contribution of PTFE (−3.7 × 10^–7^ emu g^–1^) and reduced using the molecular weight (886.45 g mol^–1^) and intrinsic diamagnetism (−462 × 10^–6^ emu mol^–1^, from Pascal’s constants^30^) appropriate for **1**·1.90Et_2_O·0.13CH_2_Cl_2_. Alternating current (ac) magnetic data were collected on the same sample and on a sample of **1*****m*** (8.36 mg) by using the same setup for frequencies (ν) between 1 Hz and 1 kHz and an exciting oscillating field amplitude of 3 Oe.

### EPR Spectroscopy

X-band (*ν* ≅ 9.40 GHz) CW-EPR spectra on solutions were recorded on a Bruker EMX spectrometer equipped with an SHQ cavity. Low-temperature measurements were obtained using a finger dewar working at 77 K. Solutions in toluene-*d*_8_/CD_2_Cl_2_ (1:1 v/v) were prepared in an Ar-filled glovebox with dioxygen and water levels below 0.5 ppm. The concentration of vanadyl ions was 1 mM for **1*****m*** and 3 mM for [VO(acac)_2_]. The EPR tubes were sealed with PTFE tape before extraction from the glovebox. X-band (*ν* ≅ 9.40 GHz) CW-EPR spectra on single crystals of **1*****h*** and **1*****m*** were recorded on a Bruker Elexsys E500 spectrometer equipped with an SHQ cavity. Low temperatures were obtained using an Oxford Instruments ESR900 continuous flow helium cryostat equipped with an Oxford Instruments ITC503 temperature controller. Crystals of **1*****h*** and **1*****m*** glued on an acetate sheet were indexed using a Bruker D8 VENTURE single-crystal diffractometer with Cu–Kα (λ = 1.54178 Å) radiation. This allowed us to identify the *c* symmetry axis and the *ab* plane in the case of **1*****h***, and the *b* axis and the *ac* plane for **1*****m***. Due to the morphology of the crystals, the identification of the relevant directions in the *ab* plane (**1*****h***) and in the *ac* plane (**1*****m***) was not possible. The crystals were then transferred to the polyethylene rod sample holder. Their orientation with respect to the magnetic field was controlled by a digital programmable goniometer (ER218PG1, Bruker BioSpin). X-band (*ν* ≅ 9.74 GHz) pulsed EPR spectra of all samples were recorded on a Bruker Elexsys E580 spectrometer equipped with a dielectric ring resonator (ER4118X-MD5) housed in a Cryogenic cryogen-free variable temperature cryostat. During the measurements, the resonator was overcoupled to minimize ringdown following the application of the microwave pulses. The electron spin echo (ESE)-detected EPR spectra were measured at *T* = 30 K using a Hahn echo sequence (π/2−τ–π–τ–echo) while sweeping the field with τ = 200 ns. Coherence times were measured using the Hahn echo sequence with incremented τ. Spin–lattice relaxation times were measured using inversion recovery sequence π−*t*_w_–π/2−τ–π–τ–echo with incremented waiting time *t*_w_ and τ = 200 ns. Rabi oscillations were measured using sequence *t*_nut_–*t*_w_–π/2−τ–π–τ–echo by incrementing the length of the nutation pulse *t*_nut_ (from 0 to 2046 ns in steps of 2 ns, or from 0 to 4092 and 4 ns increment for the highest attenuation) while keeping *t*_w_ = 4 μs and τ = 200 ns fixed. The linear dependence with respect to the applied microwave field was demonstrated by recording Rabi traces as a function of the microwave power attenuation from 0.5 to 9.5 dB. *T*_1_ and *T*_m_ measurements were fitted according to a biexponential model, described by eqs 1 and 2, respectively:
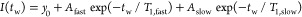
1
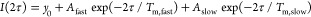
2Such a choice is well documented in the literature and is explained as follows. Regarding *T*_1_ (i.e., inversion recovery experiments), the fast component is usually attributed to spectral diffusion, whereas the slow component is usually assigned to the actual spin–lattice relaxation. Similarly, for *T*_m_ measurements (i.e., echo decay experiments), the fast component is attributed to spin diffusion while the slow component is taken to represent *T*_m_.

All simulations were performed using the EasySpin 5.2.35 package^31^ working in Matlab.

### Computational Details

Quantum chemical calculations were performed at the density functional theory (DFT) level on the different conformers of [VO(bdhb)], in the gas and solution phase (toluene, CH_2_Cl_2_, and a 1:1 v/v mixture of the two solvents). The ground electronic state molecular geometry of each species was fully optimized, using the hybrid functional M06-2X^32^ coupled with a combination of 6-311++G(d,p) (for non-d-block elements) and 6-311+G(2d) (for vanadium atoms) Pople triple-ζ basis sets.^33−41^ The geometry corresponding to the potential energy hypersurface (PEH) stationary point as obtained upon optimization was submitted to the calculation of vibrational frequencies (in harmonic approximation) to verify the nature of the stationary point; the absence of negative frequencies confirmed that a (local) minimum was reached. Along with vibrational frequencies, thermochemical quantities (at *T* = 298.15 K and *p* = 1.00 atm) and the IR intensities were also computed. Solvent effects were included by means of the SMD^42^ model, with standard values of cavitation radii. The standard values of dielectric constant and refractive index were assumed for pure solvents, whereas the model described in ref (43) was used to estimate the values for the mixture.

Using the corresponding optimized geometries, the **g** and **A** tensors were computed at the DFT level, in the gas and solution phase (toluene, CH_2_Cl_2_, and a 1:1 v/v mixture of the two solvents). Relativistic effects were included at the Zero-Order Regular Approximation (ZORA) level.^44^ Two functionals were tested, viz the pure BP86^45,46^ functional and the τ-Dependent Gradient-Corrected Correlation TPSSh functional,^47−49^ both coupled with the all-electron ZORA-def2-TZVP basis^50^ (for all atoms). The auxiliary basis for the RIJ approximation used during the relativistic case here was chosen as the appropriate SARC/J. Solvent effects were included using the conductor-like polarizable continuum model (C-PCM),^51^ using the standard values of cavitation radii (UFF with α = 1.1). The same values of dielectric constant and refractive index as used during the optimization were assumed.

For all calculations, the integration grid for the electronic density was set to 250 radial shells and 974 angular points for all of the atomic species. Accuracy for the two-electron integrals and their derivatives was set to 10^–14^ a.u. The Self-Consistent Field (SCF) algorithm used was the quadratically convergent procedure designed by Bacskay,^52^ a method which is acknowledged to be slower but more reliable than regular SCF with DIIS extrapolation. The convergence criteria for SCF were set to 10^–10^ for root-mean-square (RMS) change in density matrix and 10^–8^ for maximum change in density matrix. Convergence criteria for geometry optimizations were set to 2 × 10^–6^ a.u. for maximum force, 1 × 10^–6^ a.u. for RMS force, 6 × 10^–6^ a.u. for maximum displacement, and 4 × 10^–6^ a.u. for RMS displacement.

Molecular geometry optimizations and calculations of vibrational frequencies and thermochemical quantities were performed using GAUSSIAN G16.C01 package.^53^ The **g** and **A** tensors were computed using ORCA 5.0.2 quantum chemistry code.^54,55^ The noncovalent interactions (NCIs) were computed and depicted using a homemade code.

## Results and Discussion

### Synthesis

The procedure described by Yamamura et al. for the synthesis of [UO_2_(bdhb)]·2EtOH·0.5H_2_O^13^ and that reported by Odunola and Woods for the preparation of vanadyl complexes of 3-substituted 2,4-pentanedionates^56^ were initially tested but gave unsatisfactory results, as they afforded either intractable materials or incomplete metalation. The best results were obtained by the method of Mahroof-Tahir et al. for the preparation of vanadyl complexes of 2,2,6,6-tetramethyl-3,5-heptanedione and 3,5-heptanedione.^57^ Slight modifications were necessary to countermeasure the very low solubility of H_2_bdhb in water. Briefly, VOSO_4_·3.6H_2_O and H_2_bdhb were reacted in 1.5:1 molar proportion in a H_2_O/EtOH solvent mixture (2.5:1 v/v) at 80 °C for 4 h. This afforded a green solid typically analyzing as VO(bdhb)(EtOH)_0.35_(H_2_O)_0.35_ (yield 74%), where the occurrence of residual EtOH and H_2_O molecules was confirmed by ^1^H NMR (Figure S1) and FT-IR spectroscopy (Figures S7 and S8). Slow diffusion of Et_2_O vapors into a green solution of this crude material in CH_2_Cl_2_ gave large hexagonal rods (Figure S10a) belonging to trigonal space-group *R*3̅ and containing the centrosymmetric dimeric complex **1** (Chart 1). The formula of the compound, inferred from elemental analysis and ^1^H NMR in CDCl_3_ (Figure S2), is **1**·1.90Et_2_O·0.13CH_2_Cl_2_ (**1*****h***) (Note: the crystal phases are labeled with the crystal family symbol in Pearson notation^58^).

The crude material and crystalline **1*****h*** have similar spectroscopic properties both in the solid state and in CH_2_Cl_2_ solution. Their FT-IR spectra show minor differences related to the different lattice solvents, i.e., EtOH/H_2_O vs Et_2_O/CH_2_Cl_2_ (Figures S7 and S8). The characteristic V=O stretching frequency at 995–998 cm^–1^ is accompanied by V–O stretching band at 480–483 cm^–1^.^59^ Considering these similarities, a dimeric structure is tentatively assigned to the crude material. The UV–vis–NIR spectra in CH_2_Cl_2_ (Figure S9) display the strong π → π* absorption of the organic ligand at 304 nm (275 nm in H_2_bdhb). The characteristic electronic absorptions of vanadyl complexes are also observed at 683, 580, and ∼420 nm in the crude material, and at 683, 593, and 396 nm in **1*****h***, whose electronic spectrum is better resolved. This pattern of transitions matches well that observed for [VO(acac)_2_] in chloroform^60^ and confirms the assembly of a vanadyl complex of bdhb^2–^.

The ^1^H NMR spectra of **1*****h*** in CDCl_3_ (Figure S2) and in CD_2_Cl_2_ (Figure S4) are very similar, with paramagnetically broadened signals but very limited paramagnetic shifts, all δ values being confined between 0 and 8 ppm. In fact, as a consequence of the square-pyramidal coordination geometry and the short V=O bond, the unpaired electron of vanadyl complexes mainly occupies the metal 3d_*xy*_ orbital, whose lobes are not directed toward the donor atoms; a small spin density is consequently transferred to the organic ligand, as found in [VO(acac)_2_].^61,62^ The broad peaks in the high-field region (2–4 ppm) likely arise from the CH_2_ and CH_3_ groups of the ligand branches, with the uppermost field signal exhibiting the same δ (2.3 ppm) as the CH_3_ peak in [VO(acac)_2_].^63,64^ The somewhat narrower signals around 7 ppm are assigned to the aromatic protons of the 1,3-phenylene group.

Upon prolonged standing in their mother solution at RT, the crystals of **1*****h*** were found to convert into parallelogram-shaped crystals (Figure S10b) of a monoclinic solvatomorph with formula **1**·0.80Et_2_O·0.20CH_2_Cl_2_ (**1*****m***), as established by elemental analysis and ^1^H NMR in CDCl_3_ (Figure S3). Occasionally, thin plate-like crystals of a third solventless triclinic form (**1*****a***) were also individuated. The crystalline material obtained by conversion of the trigonal solvatomorph was analyzed through PXRD, which reproducibly demonstrated the presence of **1*****m*** and minor amounts of **1*****a***, but no residual **1*****h*** (Figure S11). In particular, QPA on the PXRD pattern obtained by the Rietveld refinement showed that the weight percentages of **1*****m*** and **1*****a*** are 84 and 16%, respectively (Figure S12).

### X-ray Crystallography

**Figure 1:**
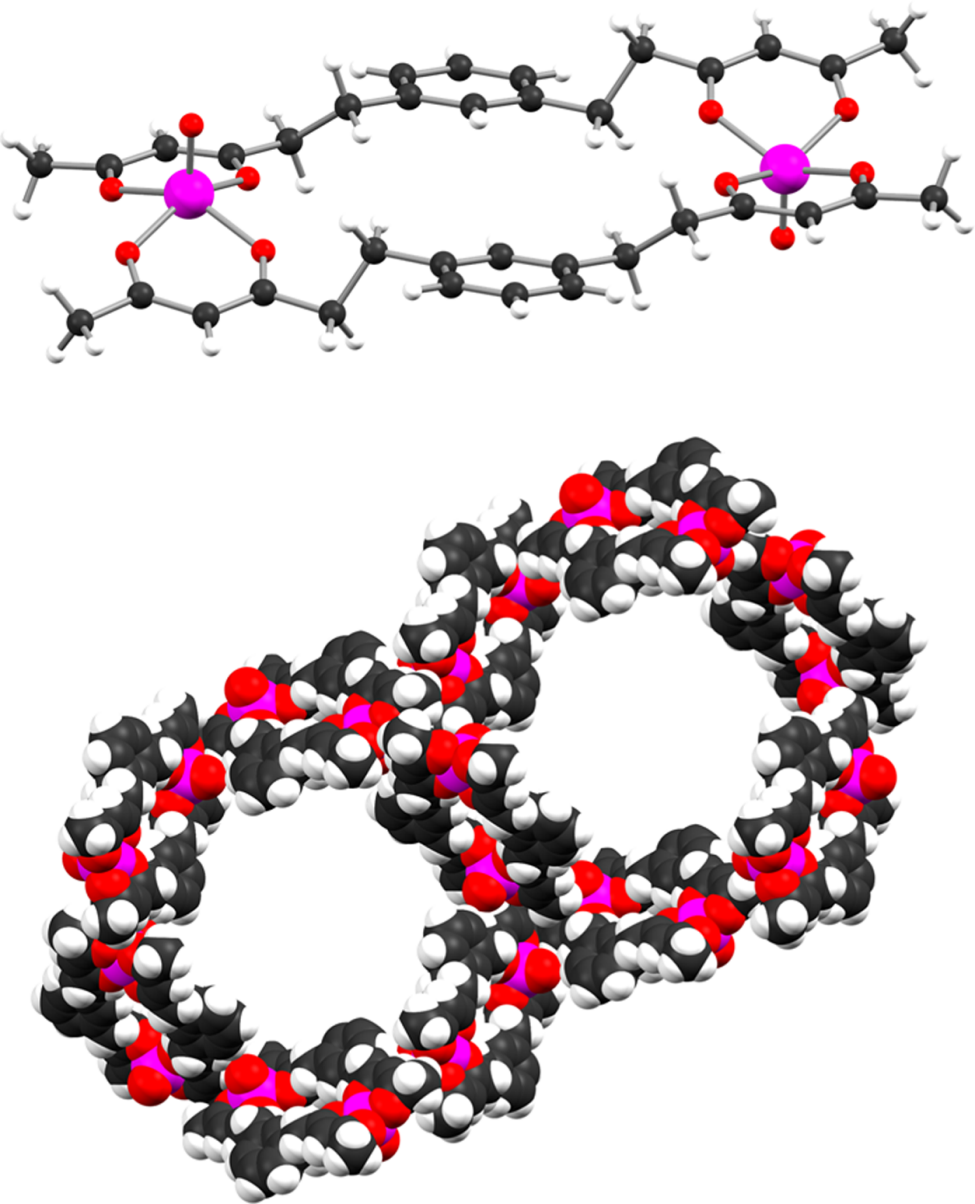
Molecular structure of **1** in **1*****h*** (top) and 3D nanoporous crystal structure of **1*****h***, viewed along the *z* axis (bottom). Color code: black (C), red (O), white (H), pink (V).

All isolated crystal phases comprise centrosymmetric dimers **1**, in which the two acac-like terminations of the bdhb^2–^ ligands bind different metal ions (Figures 1 and S13–S15). This keeps the metal ions ca. 12 Å apart from each other. The two V=O groups adopt a *trans* configuration and the bond distances and angles around the metal ions are typical for vanadyl complexes (Table 1). Overall, the molecular structure resembles that of divanadium(IV) complexes assembled using more rigid bis(β-diketonato)^65,66^ or bis-hydroxyphenylpyrazolyl^67^ ligands.

**Table 1:** Selected Structural Data for **1*****h***, **1*****m***, and **1*****a*** at 100 K

	**1*****h***	**1*****m***	**1*****a***
V···V′ (Å)^a^	11.8929(5)	11.9087(8)	12.0824(10)
V=O (Å)	1.5933(11)	1.5864(17)	1.593(2)
V–O (Å)	1.96–1.97	1.96–1.97	1.96–1.98
O=V–O (deg)	105.2–108.0	104.6–106.1	105.2–106.3
O–V–O (deg)	82.0–88.2	83.2–87.8	83.8–88.1

a V and V′ are symmetry-related by inversion through the center of the molecule.

In the crystals of **1*****h***, the platelet-like molecules stack into columns along the *z* axis (Figure S16). Within these columns, π–π stacking interactions occur between the phenyl rings of the bdhb^2–^ ligands and the six-membered chelate rings of neighboring molecules (3.62 Å distance between centroids, see Figure S17).^68^ The line joining the two metal atoms in the same molecule forms an angle of 39.6° with the *z* axis, while the V=O vectors are directed at 59.8° (or 120.2°) from *z*. The columns assemble laterally around 3_1_ (or 3_2_) axes affording a nanoporous structure with wide channels developing along the 3̅ axes (Figure 1). The closest V···V separation in the crystal [7.1488(4) Å] occurs within the array of metal centers nearest to and spiralizing around the 3-fold screw axes (Figure S18) and is considerably shorter than the intramolecular V···V distance [11.8929(5) Å]. As a relevant feature, the crystal contains a very significant amount of solvent-accessible voids (46%). Based on ^1^H NMR, the content of the channels was identified as a 94:6 mol/mol mixture of Et_2_O and CH_2_Cl_2_, whose contribution to the scattering of X-rays was removed using the SQUEEZE routine.^26^ By symmetry, the V=O groups are collinear within the same molecule, but highly noncollinear between molecules related by the 3-fold inversion axes or screws. A network of weak C–H···O hydrogen bonds involving methanide, methylene, and methyl hydrogens, with a minimum H···O separation of 2.47 Å, potentially contributes to crystal cohesion (Figures S16 and S18).

The molecular structure of the monoclinic (**1*****m***) and triclinic (**1*****a***) solvatomorphs is virtually identical to that of **1*****h*** (Table 1), but the crystal packing is much denser. The crystal lattice of **1*****m*** contains Et_2_O and CH_2_Cl_2_ molecules sharing the same crystallographic site, whereas **1*****a*** contains no crystallization solvent. In **1*****m***, dimers also pack into columns along the *x* axis and the unit cell parameter *a* corresponds to the shortest interdimer V···V distance of 7.4835(2) Å. A similar stacking of molecules related by unitary translation along the *x* axis occurs in **1*****a*** (*a* = 7.4876(4) Å), but the shortest V···V distance of 6.7109(9) Å involves molecules belonging to neighboring rows. As in the trigonal crystal form **1*****h***, π–π stacking interactions between phenyl and chelate rings are presumably operative within columns, with centroid-centroid distances of 3.56 (**1*****m***) and 3.52 Å (**1*****a***). The similar packing motif in the three crystal phases is reflected by the similar lattice periodicity along the stacking direction (*c* in **1*****h***, and *a* in both **1*****m*** and **1*****a***). By symmetry, **1*****a*** is the only crystal phase in which all V=O groups are strictly collinear.

### Solid-State Magnetic Measurements and CW-EPR Spectra

The χ_M_*T* product measured on **1*****h*** as a function of temperature (Figure S19) approaches a high-temperature value of 0.74 emu K mol^–1^, in agreement with the expectation for two uncoupled VO^2+^ units with *S* = 1/2 (0.739 emu K mol^–1^ with *g* = 1.985). χ_M_*T* starts decreasing below 30 K, indicating the existence of weak antiferromagnetic interactions in the crystal. Tentative fits using either the Bleaney–Bowers equation for two *S* = 1/2 centers (BB)^69^ or Eggert–Affleck–Takahashi equation for an antiferromagnetic regular spin-chain (AT)^70^ provided a reasonable reproduction of the experimental curve, with *J* = 0.9 ± 0.2 cm^–1^ (BB) or *J* = 0.24 ± 0.05 cm^–1^ (AT) and keeping the *g* value fixed to 1.988 (*Ĥ* = *J***Ŝ**_*i*_·**Ŝ**_*j*_). The presence of weak antiferromagnetic interactions in the crystal is confirmed by the isothermal magnetization vs field curves measured at low temperatures (Figure S20). At the highest measured field, they reach values below the expectation for a pair of noninteracting *S* = 1/2 spins with *g* = 1.988.

It is well known that the angular dependence of the EPR line width (Δ*B*_pp_) of a concentrated magnetic system strongly depends on the magnetic dimensionality of the system.^71^ Single-crystal CW-EPR spectroscopy was then used to define the dimensional magnetic behavior and to get information on the most relevant magnetic interactions within the crystal lattice of solvatomorphs **1*****h*** and **1*****m*** (we could not sort out a single crystal of triclinic solvatomorph **1*****a*** with appropriate size).

**Figure 2:**
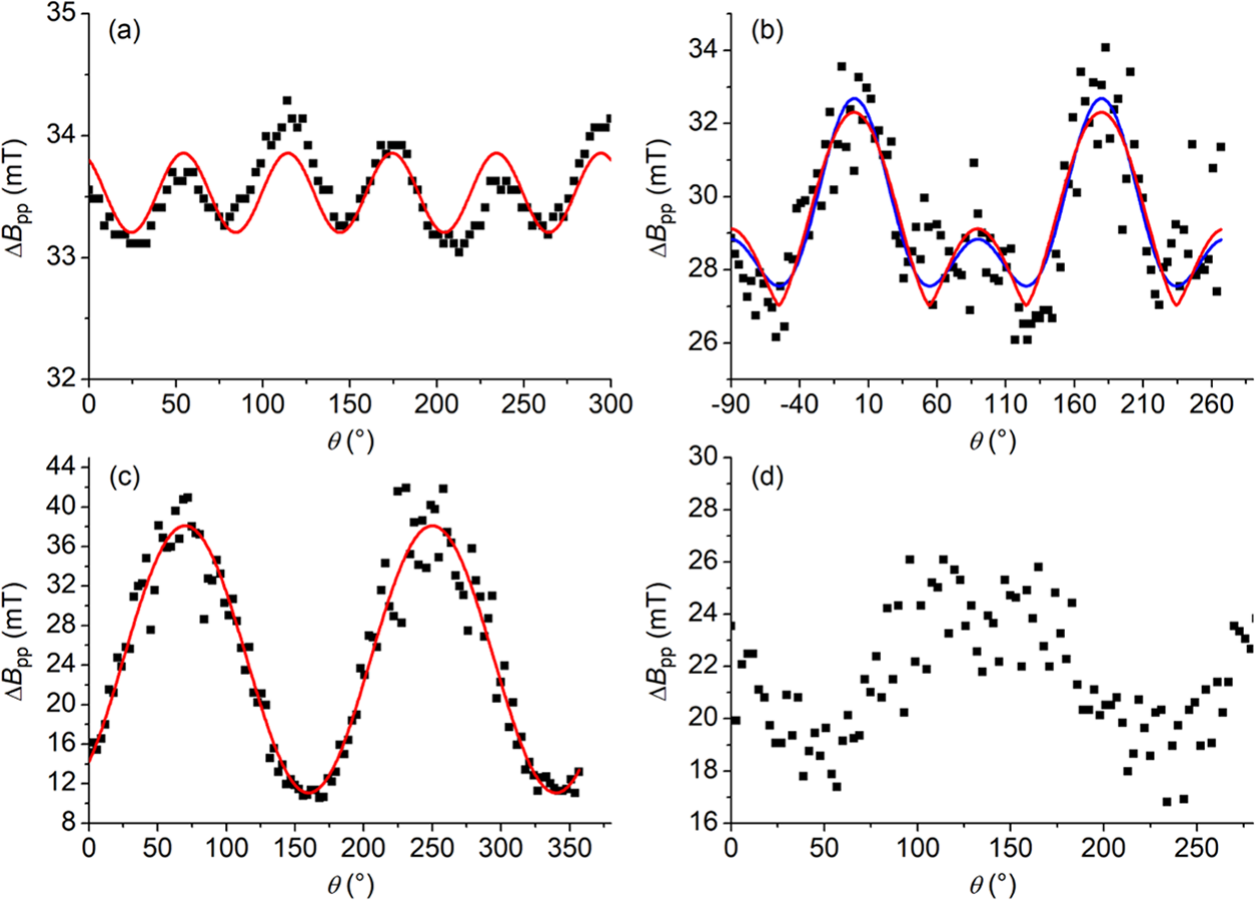
Angular dependence of the EPR line width of **1*****h*** and **1*****m*** at 50 K in different planes. (a) **1*****h***, rotation of the magnetic field in the *ab* plane and best-fit reproduction using a functional form of the type Δ*B*_pp_ = *A* + *C* [cos(6θ + ϕ)], with *A* = 33.53 ± 0.02 mT, *C* = 0.32 ± 0.03 mT, ϕ = 34 ± 5°. (b) **1*****h***, rotation of the magnetic field from the *c* axis (θ = 0°) to the *ab* plane and best-fit reproduction using a functional form of the type Δ*B*_pp_ = *A* + *C* |3  cos^2^ θ – 1|^*n*^, with *n* = 2 for the blue line (*A* = 27.6 ± 0.1 mT, *C* = 1.27 ± 0.08 mT) and *n* = 4/3 for the red line (*A* = 27.0 ± 0.2 mT, *C* = 2.1 ± 0.1 mT), expected for 2D and 1D, respectively. (c) **1*****m***, rotation of the magnetic field in the *ac* plane and best-fit reproduction using a functional form of the type Δ*B*_pp_ = *A* + *C* [1 + cos^2^(θ + ϕ)], with *A* = 65.1 ± 0.9 mT, *C* = −27.0 ± 0.6 mT, ϕ = 20.7 ± 0.6°. (d) **1*****m***, rotation of the magnetic field in an arbitrary plane containing the *b* axis.

The spectrum of **1*****h*** was recorded as a function of rotation angle, with the magnetic field applied in the crystallographic *ab* plane and from the *c* axis to the *ab* plane (Figure S21a,b). The spectrum always consists of a single, partially structured, and dipolar broadened line, centered at *g* ≈ 1.985 (see Figure S22 for an example). For the rotation of the magnetic field in the *ab* plane, the line width (Figure 2a) follows a π/3 periodicity, in agreement with the symmetry of the crystal lattice. This suggests that the broadening hides the contribution of the three magnetically inequivalent molecules in this plane. As the direction of the magnetic field is rotated from the *c* axis (θ = 0°) to the *ab* plane (Figure 2b), the angular dependence of the line width is of the type |3 cos^2^ θ – 1|^*n*^, with *n* = 2 or 4/3. This behavior is typical of low-dimensional magnetic systems and indicates that, at least along one direction, the intermolecular magnetic interactions in **1*****h*** are sizable, confirming the outcome of the magnetic susceptibility measurements. By looking at the possible interactions responsible for this type of behavior, we can single out a helix of dipolar interactions in the crystal (Figure S18) which would be compatible with *n* = 4/3.^71^ It is also evident, however, that a purely one-dimensional (1D) behavior is far from being achieved, since many other interchain dipolar interactions can be identified.

In agreement with this interpretation, the observed behavior is completely different for the monoclinic solvatomorph **1*****m***, where the helix of dipolar interactions is absent. In this case, the angle-dependent spectra were obtained by rotating the magnetic field in the *ac* plane and in a plane containing the *b* axis (Figure S21c,d). The analysis of the line width as a function of rotation angle (Figure 2c,d) evidences a clear π periodicity with an angular dependence of the type (1 + cos^2 ^θ) for the rotation in the *ac* plane. This type of dependence indicates the relevance of nonsecular terms (including anisotropic exchange)^71^ and is consistent with the increased interactions expected on reducing the intermolecular distance within the lattice, as observed by X-ray diffraction.

**Figure 3:**
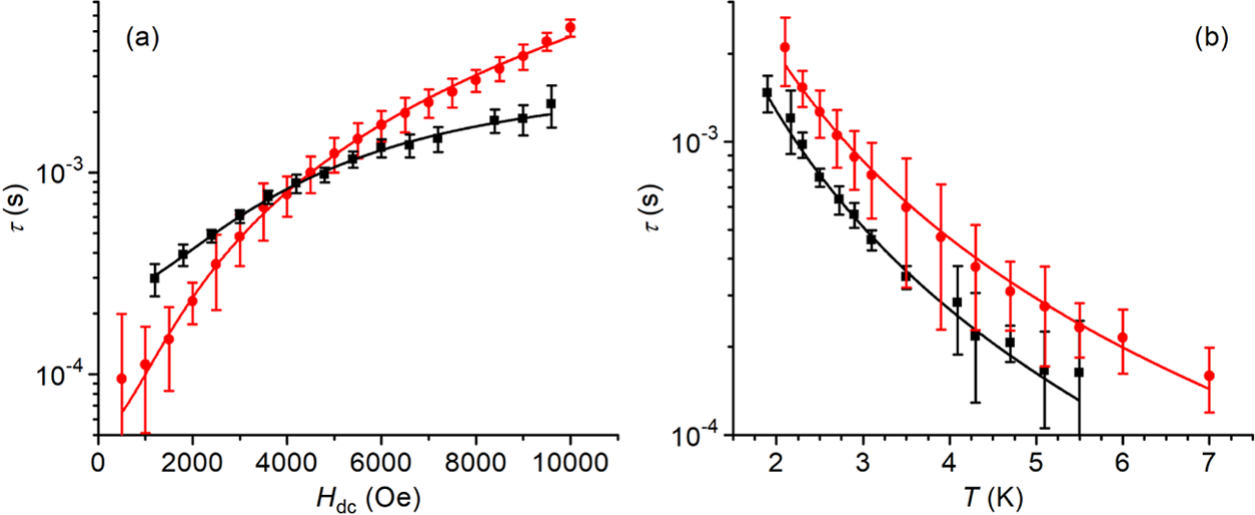
Spin–lattice relaxation time of **1*****h*** (black squares) and **1*****m*** (red circles). (a) Field dependence of τ at 1.9 K for both derivatives. (b) Temperature dependence of τ at *H*_dc_ = 7200 Oe (**1*****h***) and 7500 Oe (**1*****m***). The solid curves provide the best fit to the data using eqs 3 and 4 with the parameters gathered in Table 2.

The spin–lattice relaxation of both **1*****h*** and **1*****m*** was evaluated in nonresonant conditions by variable-frequency ac susceptibility measurements performed as a function of field and temperature. The magnetic susceptibility of **1*****h*** shows no out-of-phase component (χ_M_’’) in zero field at 1.9 K, as expected for *S* = 1/2 spins. As often reported for this type of systems,^8,67,72−74^ a nonzero χ_M_’’ value appears upon application of a small dc field (*H*_dc_). At *H*_dc_ = 1200 Oe and 1.9 K, the maximum of χ_M_’’(ν) falls at the highest extreme of the investigated frequency window (1 Hz to 1 kHz). On increasing *H*_dc_ up to 9600 Oe, the maximum monotonously shifts to a lower frequency, indicating a slowing down of the relaxation (Figure S23). To obtain a more quantitative determination of the spin–lattice relaxation time (τ), we simultaneously fitted the χ_M_’(ν) and χ_M_’’(ν) curves to a generalized Debye model^75^ using a previously developed software (Table S2).^76^ The resulting field dependence of τ confirms that on increasing field the relaxation time increases (Figure 3a). The fact that at high field the relaxation time is still increasing indicates that the effect of spin–spin interactions is relatively strong; at the same time, the direct process, which should reduce the relaxation time on increasing the field, does not seem to play a major role here. Given that for fields higher than 7200 Oe the distribution parameter α reaches quite large values (Table S2), we selected this specific dc field to investigate the temperature dependence of τ. This allowed us to observe a maximum in the χ_M_’’(ν) curve and to determine τ values up to 5.5 K (Figures 3b and S24, and Table S2).

A qualitatively similar behavior was observed for **1*****m*** (Figures S2 and S24, and Table S3). However, while the low-field relaxation rate is faster in **1*****m*** than in **1*****h***, for fields higher than 4500 Oe, the monoclinic solvatomorph relaxes slower (Figure 3a). Consequently, at *H*_dc_ = 7500 Oe, the χ_M_’’(ν) maximum occurs within the experimental frequency window up to 7 K (Figures 3b and S24).

These differences are consistent with the different crystal structures of the two compounds. **1*****m*** is indeed characterized by a more compact packing and stronger spin–spin interactions as compared with nanoporous **1*****h***. Its relaxation is thus faster than that of **1*****h*** in low fields. Once the spin–spin interactions are overcome by the applied field, the more compact and stiffer lattice of **1*****m*** results in a somehow slower spin–lattice relaxation. Given that in both solvatomorphs the contribution of the direct mechanism is clearly weak in the investigated field range, the τ vs *H*_dc_ curves were fitted to eq 3, which accounts for a temperature-independent, quantum-tunneling-like behavior^77^
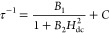
3The best reproduction of the two curves was obtained using the parameters reported in Table 2.

**Table 2:** Spin–Lattice Relaxation Parameters in **1*****h*** and **1*****m***^a^

	*B*_1_ (s^–1^)	*B*_2_ (Oe^–2^)	*C* (s^–1^)	*A* (s^–1^ K^–*n*^)	*n*
**1*****h***	3775 ± 212	(2.0 ± 0.2)×10^–7^	322 ± 22	165 ± 11	2.24 ± 0.06
**1*****m***	18820 ± 3875	(8.8 ± 1.9)×10^–7^	0.5^b^	113 ± 6	2.11 ± 0.04

a Obtained by fitting the field- and temperature-dependent relaxation times with eqs 3 and 4, respectively.

b Held fixed.

On the other hand, the temperature dependence of τ in both derivatives could be reproduced by simply assuming a Raman-like behavior
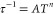
4with a value of *n* close to 2. While this can suggest^78^ the occurrence of a phonon bottleneck behavior, we refrain from any conclusions, as it would require a much deeper experimental analysis,^79^ which is beyond the scope of this work.

### Solution Structure of **1**

**Figure 4:**
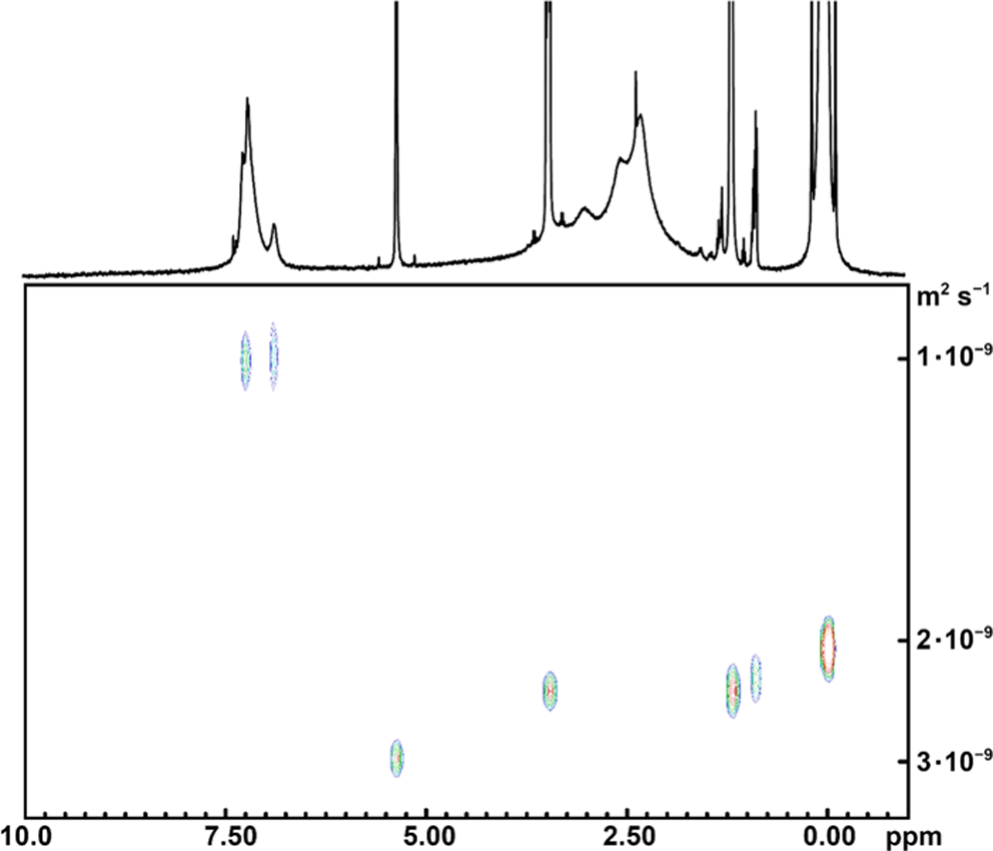
^1^H DOSY spectrum of **1*****h*** in CD_2_Cl_2_ (400.13 MHz, 298 K). The narrow peaks are from CH_2_Cl_2_ (5.33 ppm), Et_2_O (3.43 and 1.15 ppm), pump oil (1.28 and 0.88 ppm), residual proton impurities in the solvent (5.32 ppm), and TMS (0.00 ppm) which was added as an internal reference for the normalization of diffusion coefficients, following the procedure described by Stalke et al.^20^ Processing parameters (TopSpin 4.3.0^17^): SI = TD, LB = 1.00 Hz.

Since **1** does not ionize under electrospray ionization–mass spectrometry (ESI-MS) conditions, its solution structure was probed by DOSY. The DOSY technique allows to measure the diffusion coefficient (*D*) of a solute, which can be used to estimate its molecular weight (*MW*) through state-of-the-art external calibration curves (ECCs).^20,80^ DOSY analysis is usually challenging for paramagnetic compounds but is often successful when NMR signals do not undergo large paramagnetic shifts. However, another important parameter is the *w*_1/2_/*T*_1_ ratio, where *w*_1/2_ is the peak width and *T*_1_ is the longitudinal relaxation time.^80^ In the ^1^H DOSY spectrum of **1*****h*** in CD_2_Cl_2_ (Figure 4), only the two resonances around 7 ppm gave measurable responses with similar *D* values (1.1 × 10^–9^ m^2^ s^–1^ after averaging and normalization^20^). Using the parameters of ECC_DSE_ and ECC_MERGE_ provided in ref (20), the estimated *MW* values are 380 ± 110 and 412 ± 129 g mol^–1^, respectively. These estimates are much closer to the *MW* of the monomeric species [VO(bdhb)] (**1**′, 367.29 g mol^–1^) than to the *MW* of dimeric **1** (734.58 g mol^–1^), suggesting a possible structural rearrangement upon dissolution (Chart 1). It is well recognized that the *MW* of a metal-containing species can be underestimated when using ECCs based on organic molecules like ECC_DSE_ and ECC_MERGE_.^80,81^ Therefore, we tested an additional ECC specific for species containing 3d metals.^80^ The calculation afforded *MW* = 493 ± 215 g mol^–1^, a value again more consistent with monomeric **1**′ (see Figure S5). As a further proof, DOSY analysis was conducted on a CD_2_Cl_2_ solution of the free H_2_bdhb proligand (*MW* = 302.36 g mol^–1^), yielding *D* = 1.2 × 10^–9^ m^2^ s^–1^ (Figure S6). This value is only slightly larger than the diffusion coefficient of the vanadyl complex and leads to estimated *MW*s of 362 ± 105 and 391 ± 122 g mol^–1^ using ECC_DSE_ and ECC_MERGE_ parameters,^20^ respectively. This clearly indicates that the vanadyl complex has a similar size to H_2_bdhb in CD_2_Cl_2_ solution.

**Figure 5:**
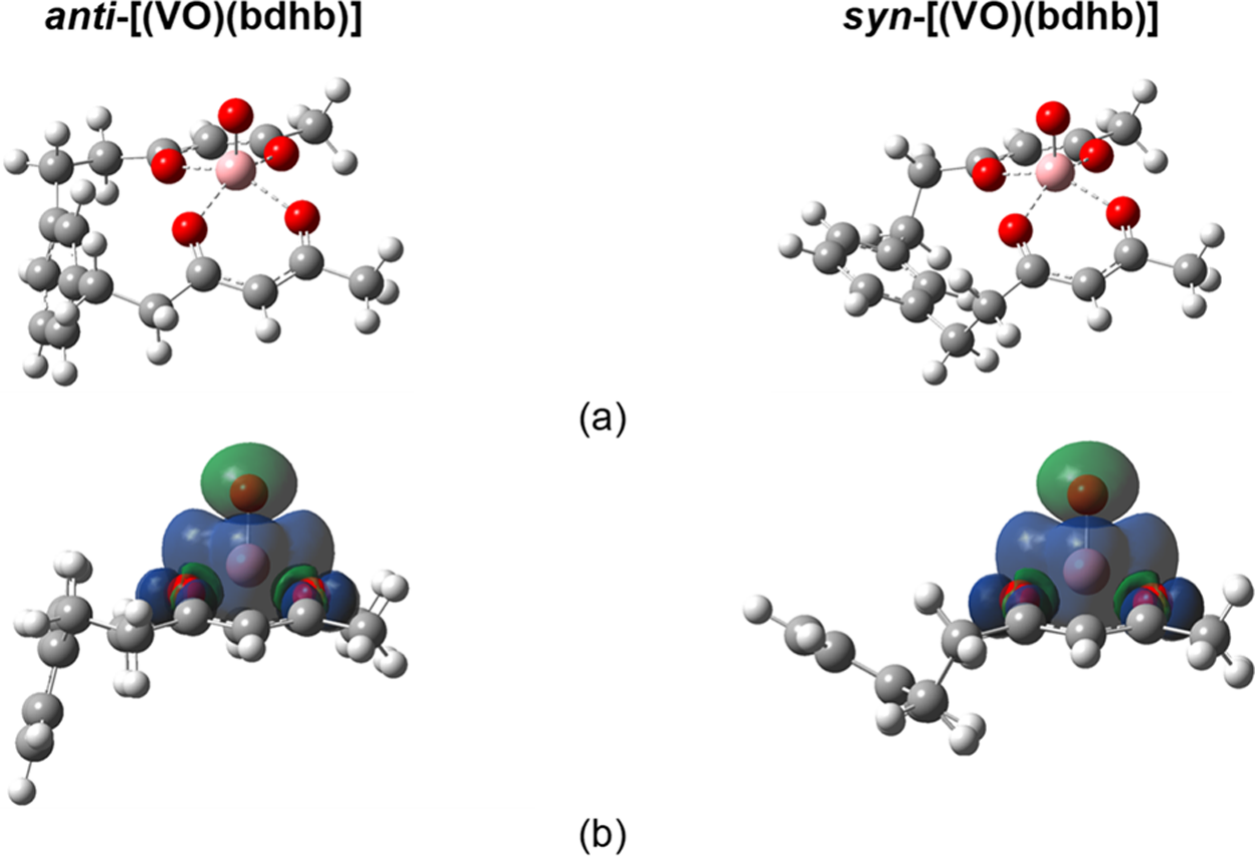
(a) Geometry of *anti*- and *syn*-[VO(bdhb)] conformers, optimized in the gas phase and (b) their spin densities (blue = positive; green = negative; |isovalue| = 4 × 10^–4^ a.u.). Legend of colors: white (H), gray (C), red (O), and pink (V).

The molecular structure of **1**′ was optimized at the DFT level using the hybrid functional M06-2X combined with a mixed basis set consisting of 6-311++G(d,p) (for non-d-block elements) and 6-311+G(2d) (for vanadium atoms) Pople triple-ζ basis sets, both in the gas phase and surrounded by implicit solvation. Two conformers (*anti* and *syn*) were individuated differing in the relative orientation of the 1,3-phenylene and V=O groups (Figure 5a). The geometries of *anti*- and *syn*-[VO(bdhb)] differ from each other also for the values of C–O (1.268–1.279, and 1.272–1.275 Å, respectively) and V–O bond distances (1.976–1.994, and 1.981–1.985 Å, respectively), whereas V=O bond length shows a negligible difference in the two conformers (1.559 and 1.560 Å, respectively).

Energy wise, the *anti* conformer is predicted to be more stable than the *syn* conformer both in the gas phase (4.9 kJ mol^–1^) and in solution (2.9–4.6 kJ mol^–1^), as reported in Table S6. This energy difference is explained on the basis of the intracomplex noncovalent interactions (NCIs; Figure S26). Both *anti-* and *syn*-[VO(bdhb)] show the presence of attractive intracomplex NCIs established between the two β-diketonato O atoms closer to the phenyl ring and two C atoms of the phenyl ring. However, NCIs in the *syn* conformer are weaker than those in the *anti* conformer, due to the larger distance. The energy differences are not large enough to let to neglect the *syn* conformer, whose population ranges from 12.2 to 23.8% in the set conditions.

**Figure 6:**
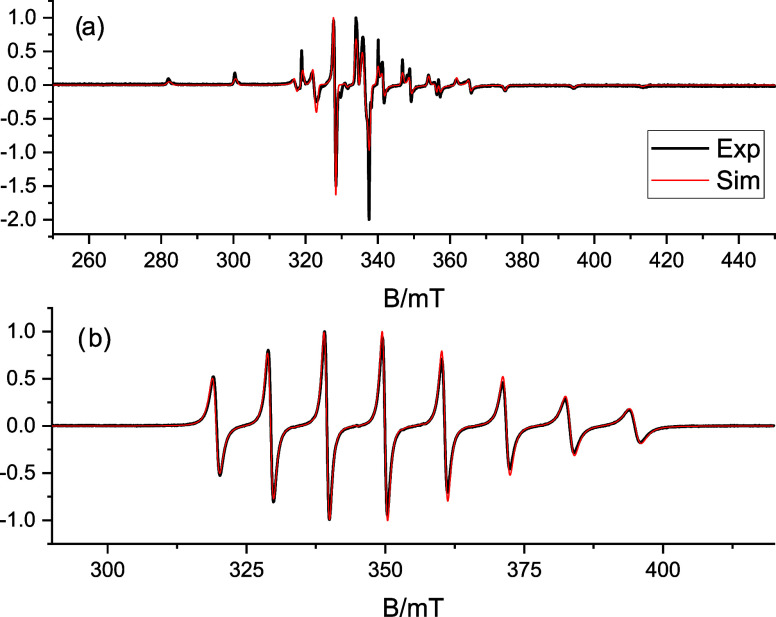
Experimental (black) and simulated (red) X-band CW-EPR spectra of a solution of **1*****m*** in toluene-*d*_8_/CD_2_Cl_2_ (1:1 v/v), recorded at 77 K (a) and RT (b).

CW-EPR spectra at X-band frequency were recorded at 77 K and RT on a solution of **1*****m*** in toluene-*d*_8_/CD_2_Cl_2_ (1:1 v/v). The observed powder pattern (Figure 6) is characteristic of an isolated and monomeric vanadyl complex in which the unpaired electron is confined in the 3d_*xy*_ vanadium orbital (*S* = 1/2) and interacts with the nuclear spin of ^51^V (*I* = 7/2, natural abundance = 99.75%).

The spectra were analyzed^31^ using the spin-Hamiltonian (SH) in eq 5
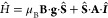
5where collinear **g** and **A** tensors were assumed. The best simulations and the corresponding SH parameters are shown in Figure 6 and Table 3, respectively. The parameters match those previously found in other vanadyl complexes displaying axial or quasi-axial symmetry.^8,82^ The spectra were recorded again after storing the solution for 6 months in an air-free environment and were found unchanged, indicating complete stability in solution.

**Table 3:** Spin-Hamiltonian Parameters from CW-EPR Spectra and DFT Calculations^a^

	*g*_*x*_	*g*_*y*_	*g*_*z*_	|*A*_*x*_|	|*A*_*y*_|	|*A*_*z*_|	τ_r_
**1*****m***^b^	1.9834	1.9775	1.9451	170	190	510	3.95 × 10^–11^
[VO(acac)_2_]^b^	1.9812	1.9783	1.9452	174	189	513	2.26 × 10^–11^
**1**′ (DFT, gas phase)^c^	1.99068	1.98826	1.96879	146.7	166.9	459.0	
**1**′ (DFT, toluene)^c^	1.99047	1.98773	1.96967	137.9	160.0	451.1	
**1**′ (DFT, CH_2_Cl_2_)^c^	1.99010	1.98683	1.97097	125.1	150.7	439.6	
**1**′ (DFT, mixture)^c^^,^^d^	1.99030	1.98728	1.97048	130.3	154.0	444.3	

a Hyperfine values are in MHz and τ_r_ in s.

b From CW-EPR spectra in toluene-*d*_8_/CD_2_Cl_2_ (1:1 v/v) at 77 K; the estimated errors are ±0.0002 and ±5 MHz for the **g** and **A** principal components, respectively.

c Average values over the *syn* and *anti* conformers, calculated using TPSSh functional; all **A** principal components are negative.

d Toluene/CH_2_Cl_2_ (1:1 v/v).

The rigid limit SH parameters obtained from the simulation of the frozen solution spectrum (Figure 6a) were used to simulate the motionally averaged (fluid solution) CW spectrum (Figure 6b), assuming an isotropic tumbling governed by Brownian motion. Under these circumstances, the only parameter necessary to characterize the speed of tumbling is the rotational correlation time, which was estimated as τ_r_ = 3.95 × 10^–11^ s (lower limit, τ_r_ = 3.37 × 10^–11^ s; upper limit, τ_r_ = 4.45 × 10^–11^ s). This value is in line with that obtained for [VO(acac)_2_] under similar experimental conditions (τ_r_ = 2.26 × 10^–11^ s, see Supporting Note 1, Table 3, and Figure S25). We now assume that the rotational correlation time τ_r_ can be approximated by the quasi-hydrodynamic relation:^83^
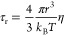
6where *k*_B_ is the Boltzmann constant (J K^–1^), *T* is the absolute temperature (K), and η is the dynamic viscosity of the solvent. Since we work with a toluene-*d*_8_/CD_2_Cl_2_ solution (1:1 v/v), the RT η values of toluene (0.560 mPa s) and CH_2_Cl_2_ (0.413 mPa s)^84^ are averaged to yield η = 0.49 mPa s. In this way, a hydrodynamic radius (*r*) of the order of 0.43 ± 0.02 nm is estimated. The value is slightly larger than the corresponding value obtained for [VO(acac)_2_] using the same approximation (*r* = 0.36 ± 0.02 nm, see Supporting Note 1). This is consistent with the molecular structure of **1**′ and indicates the formation of monomeric species in solution.

The spin densities of *anti-* and *syn*-[VO(bdhb)] evaluated by DFT are displayed in Figure 5b. They do not show remarkable differences in the two conformers and appear to be mainly localized about the V=O group, with positive/negative value about the V/O atom. The computed principal values of the **g** and **A** tensors (Table S7) are virtually the same in the two conformers. They are barely sensitive to the assumed phase and in fair agreement with the experimental data, with a calculated hyperfine coupling strength about 17% lower, on average, than observed (Table 3).

### Pulsed EPR Spectroscopy

**Figure 7:**
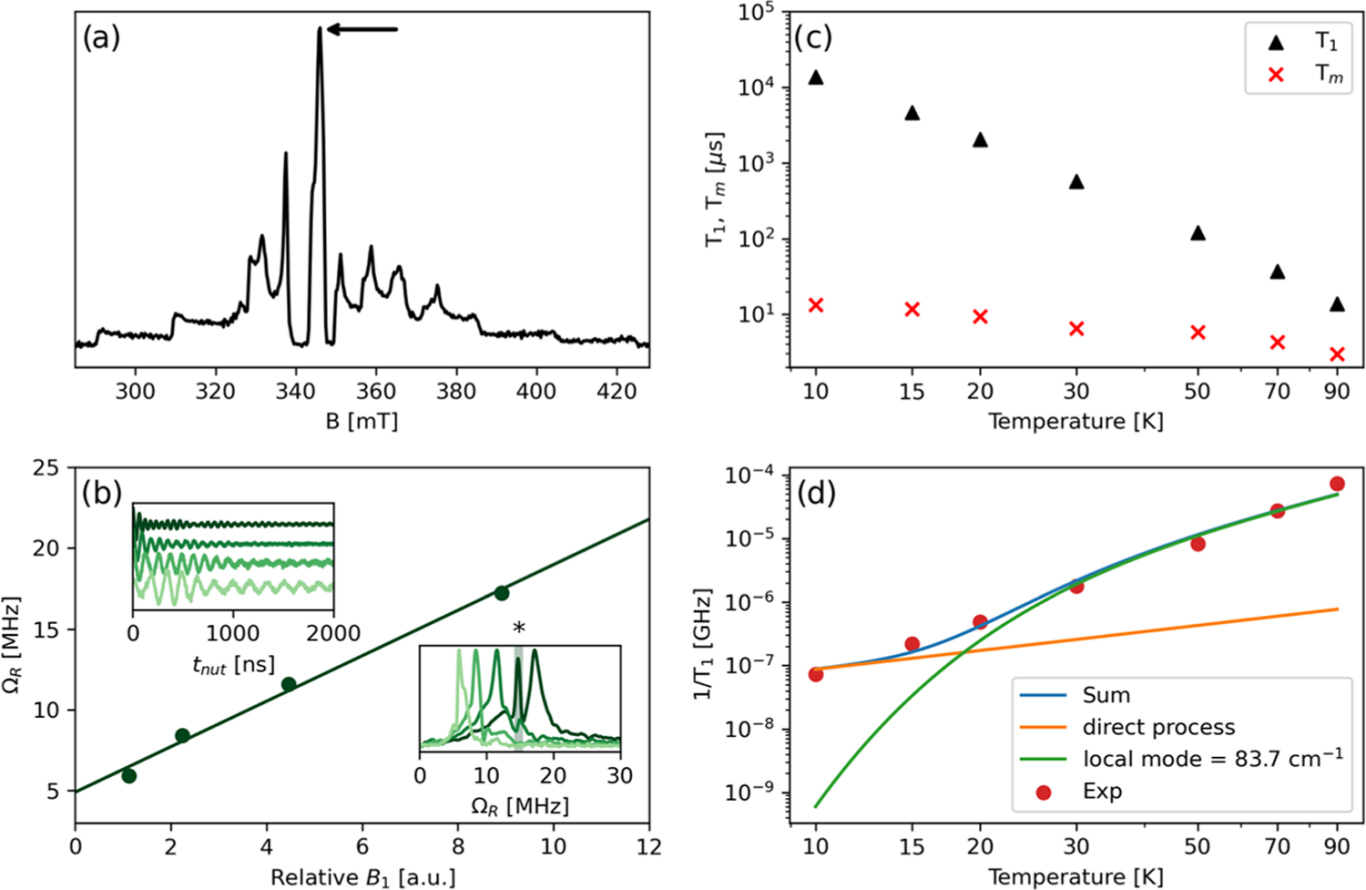
Results of X-band pulsed EPR experiments on a solution of **1*****m*** in toluene-*d*_8_/CD_2_Cl_2_ (1:1 v/v). (a) ESE-detected EPR spectrum recorded at 30 K. (b) Dependence of the Rabi frequency (*Ω*_R_) on the relative intensity of the oscillating field **B**_1_ at 70 K; the upper and lower insets show the Rabi (nutation) oscillation as a function of **B**_1_ strength in time and frequency domains, respectively; the asterisk indicates the proton Larmor frequency observed under the Hartmann–Hahn condition (|γ_e_**B**_1_|=|γ_n_**B**_1_|). (c) Temperature dependence of *T*_1_ and *T*_m_; only the slow component of the biexponential fit is plotted. (d) Fit of the temperature dependence of *T*_1_ using eq 7. All experiments were performed at the external magnetic field position indicated by the arrow in Figure 7a.

While dimeric paramagnetic systems are actively investigated as potential 2-qubit quantum gates (*qugates*),^9^ complex **1** lacks one fundamental feature to act as a qugate, namely, the individual addressability of the two paramagnetic centers, which for homonuclear systems requires both magnetic anisotropy and noncollinear magnetic axes. For this reason, pulsed EPR experiments were only conducted in frozen toluene-*d*_8_/CD_2_Cl_2_ (1:1 v/v) solution to ascertain the viability of **1**′ as a possible qubit. Such measurements entail the quantification of the relaxation times as a function of temperature and the possibility of coherently manipulating the spin state of the qubit. As a reference, the ESE-detected EPR spectrum recorded at 30 K on the same solution of **1*****m*** probed by CW-EPR is reported in Figure 7a and corresponds to the integrated CW-EPR spectral pattern (Figure 6a). The spin relaxation properties were studied by temperature-dependent echo decay (coherence time, *T*_m_) and inversion recovery experiments (spin–lattice relaxation time, *T*_1_) in the temperature interval between 10 and 90 K and are reported in Figure 7c.

*T*_m_ values were extracted by fitting the experimental decay traces using a biexponential function, whereby only the slow component of the biexponential fit is plotted (Table S4). A *T*_m_ value of the order of 13.4 μs is recorded at 10 K, which drops to about 3.0 μs at 90 K (Figure 7c); a similar behavior was reported for other VO^2+^-based molecular qubits.^82,85^*T*_1_ inversion recovery traces were similarly fitted with a biexponential function and the slow component is plotted in Figure 7c as a function of temperature. A *T*_1_ value of about 13.7 ms is observed at 10 K, which decreases to about 13.7 μs at 90 K. Therefore, at 90 K complex **1**′ approaches the high-temperature limit where *T*_1_ ≈ *T*_m_. To gain some microscopic insight into the causes of spin–lattice relaxation, the temperature dependence of *T*_1_ was modeled considering a direct mechanism of relaxation at low temperatures and a local vibrational mode responsible for the high-temperature relaxation, according to eq 7.
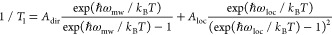
7In eq 7, the first term represents the direct process (ω_mw_ is the Zeeman frequency, with ω_mw_/2π = 9.74 GHz), while the second term accounts for a Raman process promoted by an optical mode of frequency ω_loc_. This model fairly reproduces the temperature dependence of *T*_1_ (Figure 7d) providing the frequency of a potential low-frequency local vibrational mode with ℏω = 83.7 cm^–1^ (Table S5), in close similarity with a recent study by some of us.^82^

To demonstrate the possibility of coherent spin manipulation, nutation experiments were performed at different microwave powers at 70 K (Figure 7b) and Rabi oscillations were clearly observed with the expected linear dependence of the Rabi frequency (*Ω*_R_) on the microwave attenuation (Figure 7b).

## Conclusions

The 1:1 adduct of oxovanadium(IV) with bis(β-diketonato) ligand bdhb^2–^ displays a dual structure in the crystalline state and in solution. In the crystalline state, the two β-diketonato functions of each bdhb^2–^ ligand are bound to two different metal ions, yielding dimeric molecules [(VO)_2_(bdhb)_2_] (**1**) (Chart 1). This structural motif is present in all crystalline phases isolated so far, namely, a nanoporous trigonal phase (**1*****h***) containing 46% of solvent-accessible voids, and two more tightly packed phases (monoclinic solvatomorph **1*****m*** and solventless triclinic **1*****a***). In both **1*****h*** and **1*****m***, quantum tunneling and Raman processes contribute to slow magnetic relaxation at low temperatures, which is only detectable under a static magnetic field. In the three crystal structures, the intramolecular V···V separations (11.9–12.1 Å) are significantly longer than interdimer V···V contacts (6.7–7.5 Å), causing magnetic properties to be solvatomorph-dependent. The effect was clearly evidenced by single-crystal CW-EPR studies, which showed that **1*****h*** has a predominantly 1D magnetic behavior, whereas **1*****m*** has a more complex behavior due to the increased magnetic interactions.

Solutions in organic solvents (CD_2_Cl_2_ or toluene-*d*_8_/CD_2_Cl_2_) however contain monomeric [VO(bdhb)] (**1**′) complexes, where the four β-diketonato oxygen donors are bound to the same metal ion affording a quasi-macrocyclic structure (Chart 1), as established by combined use of ^1^H DOSY experiments, CW-EPR spectra, and DFT optimizations. These isolated oxovanadium(IV) complexes exhibit a coherent spin dynamics in a frozen toluene-*d*_8_/CD_2_Cl_2_ matrix, with *T*_1_ = 14 ms and *T*_m_ = 13 μs at 10 K, and detectable Rabi oscillations at 70 K, as measured by pulsed EPR.

Since the stacking of dimers into rows is a common feature of crystal packing in the three solvatomorphs, we contend that intermolecular interactions may contribute to the observed dimerization. The rearrangement into monomeric species in solution may instead be partly or entirely driven by the associated entropy increase.

Complex **1**′ provides a new example of a vanadyl-based molecular qubit exhibiting a desirable but rare combination of features, namely: (i) a single bis-chelating ligand; (ii) a neutral charge; (iii) nuclear spin-free O donors; (iv) suitability for incorporation as a quantum sensor into more complex architectures (e.g., by functionalization at the *para*-aromatic position).^86,87^ The observed assembly of dimeric structures, albeit confined to the crystalline state, might be easily countermeasured by the cyclization reaction described by Alberts and Cram,^11,12^ a direction we are currently working in.

## Supporting Information Available

NMR, FT-IR, and UV–vis–NIR spectra; DOSY figures; additional figures and tables on X-ray crystallographic analysis; dc and ac magnetic data; and additional details on EPR spectra and DFT calculations (PDF)
Cartesian atomic coordinates of DFT-optimized structures both in the gas phase and surrounded by implicit solvation (TXT)
