# Supplementary material for: Dual Structure of a Vanadyl-Based Molecular Qubit Containing a Bis(β-diketonato) Ligand
Source: Inorg Chem. 2024 Apr 15;63(17):7912–25. doi: 10.1021/acs.inorgchem.4c00834 (PMC11391581; doi:10.1021/acs.inorgchem.4c00834)
Supplement: Supplementary file 1 — ic4c00834_si_001.pdf [file ic4c00834_si_001.pdf]

Dual structure of a vanadyl-based molecular qubit containing a bis(β-diketonato) ligand

Manuel Imperato,[a,b,#] Alessio Nicolini,[a,#] Matteo Boniburini,[a] Daniele Sartini,[c] Enrico Benassi,[b] Mario Chiesa,[d] Lara Gigli,[e] Yu-Kai Liao,[d] Arsen Raza,[c] Enrico Salvadori,[d] Lorenzo Sorace,[c] and Andrea Cornia[a,*]

[a]Dipartimento di Scienze Chimiche e Geologiche e UdR INSTM, Università degli Studi di Modena e Reggio Emilia, via G. Campi 103, 41125 Modena, Italy. E-mail: acornia@unimore.it

[b]Dipartimento di Scienze Fisiche, Informatiche e Matematiche, Università degli Studi di Modena e Reggio Emilia, via G. Campi 213/A, 41125 Modena, Italy

[c]Dipartimento di Chimica "Ugo Schiff" e UdR INSTM, Università degli Studi di Firenze, via della Lastruccia 3, 50019 Sesto Fiorentino (FI), Italy

[d]Dipartimento di Chimica e NIS Centre, Università degli Studi di Torino, via P. Giuria 7, 10125 Torino, Italy

[e]Elettra-Sincrotrone Trieste S.C.p.A., Strada Statale 14 - km 163.5 in AREA Science Park, 34149 Basovizza (TS), Italy

SUPPORTING INFORMATION

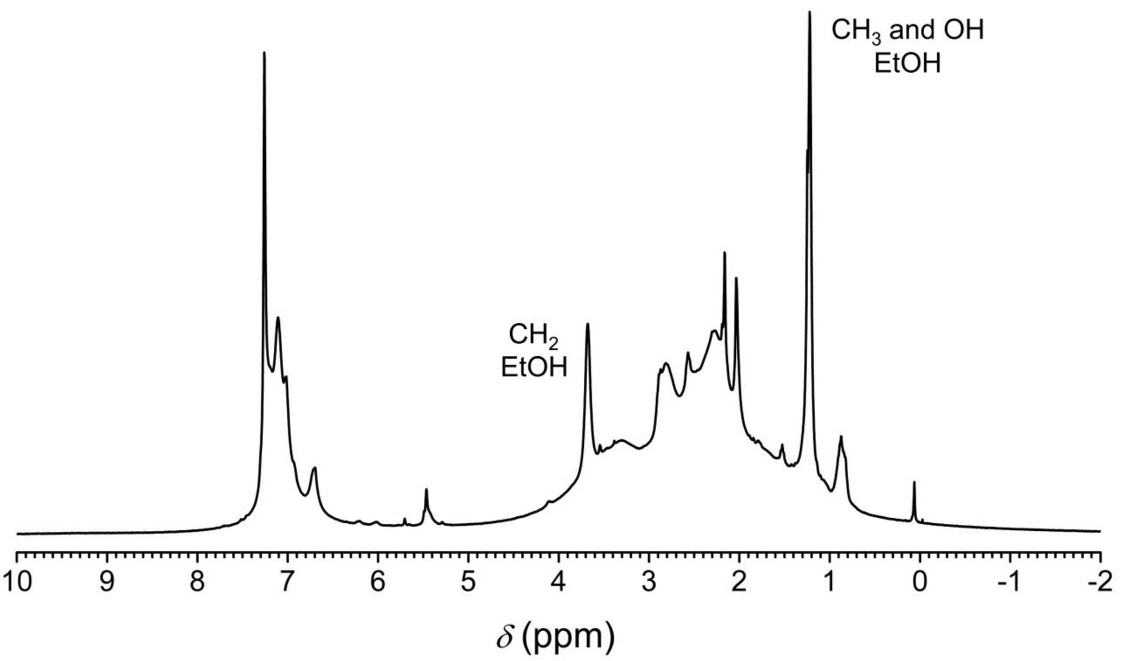

Figure S1. ^{1}H-NMR spectrum of the crude VO(bdhb)(EtOH)$_{0.35}$(H$_2$O)$_{0.35}$ material in CDCl$_3$ (400.13 MHz, 298 K). The main narrow peaks are from EtOH (3.68 and 1.22 ppm) and residual proton impurities in the solvent (7.26 ppm). Processing parameters (TopSpin 4.3.0[1]): SI = TD, LB = 1.00 Hz.

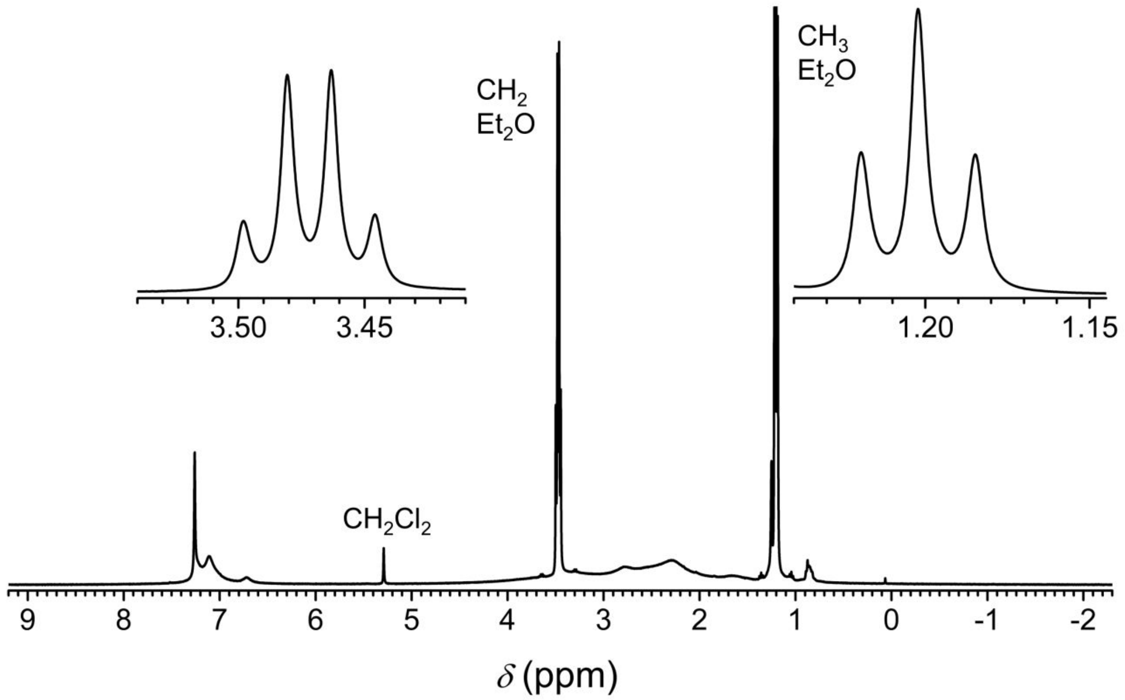

Figure S2. ^{1}H-NMR spectrum of **1*h*** in CDCl$_3$ (400.13 MHz, 298 K). The narrow peaks are from CH$_2$Cl$_2$ (5.29 ppm), Et$_2$O (3.47 and 1.20 ppm), and residual proton impurities in the solvent (7.26 ppm). The CH$_2$ signals of Et$_2$O and CH$_2$Cl$_2$ have a 4.00:0.13 area ratio, indicating a Et$_2$O/CH$_2$Cl$_2$ molar ratio of 15, in good agreement with the value obtained from elemental analysis (14.6). Processing parameters (TopSpin 4.3.0[1]): SI = TD, LB = 0.30 Hz.

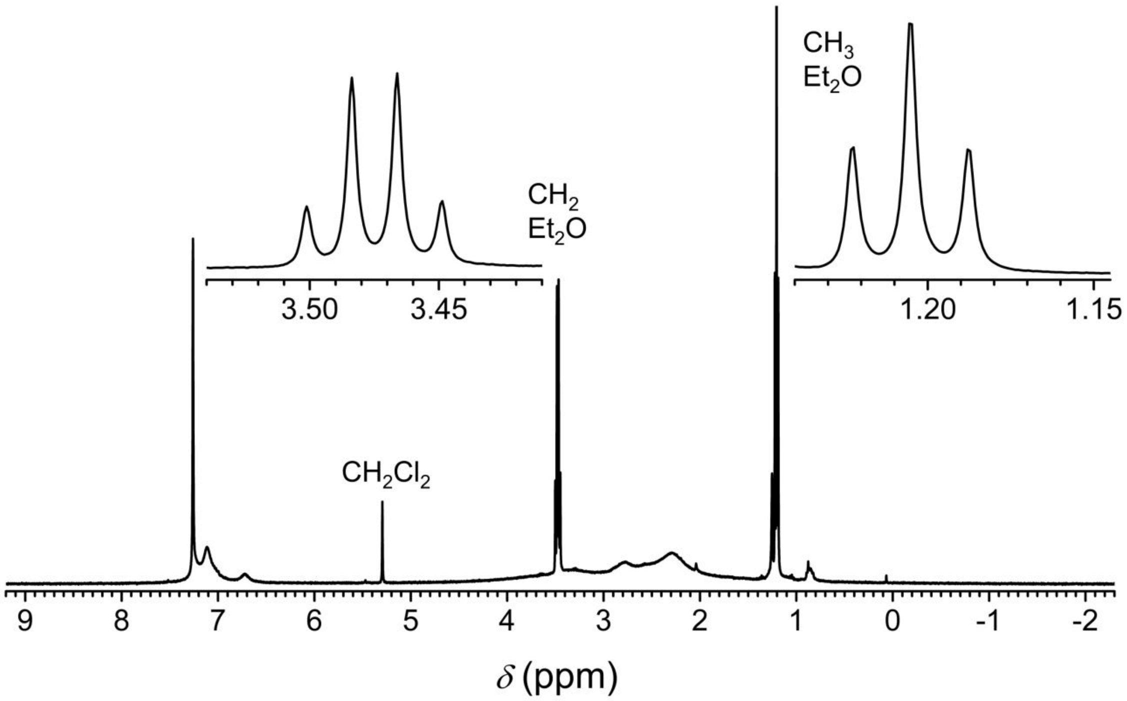

Figure S3. ^{1}H-NMR spectrum of **1*m*** in $CDCl_3$ (400.13 MHz, 298 K). The narrow peaks are from CH_2Cl_2 (5.30 ppm), Et_2O (3.47 and 1.21 ppm), and residual proton impurities in the solvent (7.26 ppm). The CH_2 signals of Et_2O and CH_2Cl_2 have a 4.00:0.52 area ratio, indicating a Et_2O/CH_2Cl_2 molar ratio of 3.8, in good agreement with the value obtained from elemental analysis (4.0). Processing parameters (TopSpin 4.3.0[1]): SI = TD, LB = 0.30 Hz.

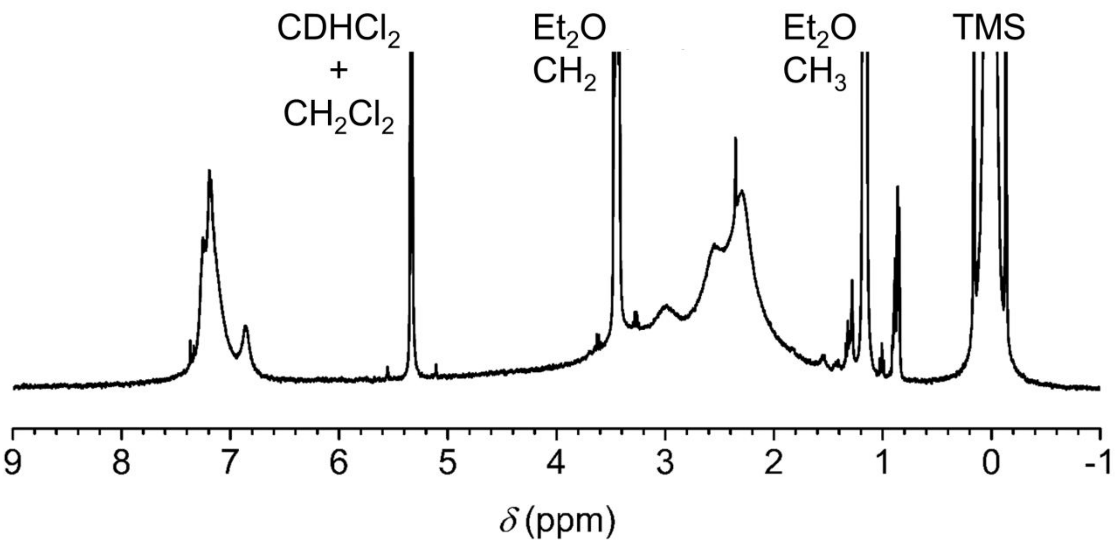

Figure S4. ^{1}H-NMR spectrum of **1*h*** in CD_2Cl_2 (400.13 MHz, 298 K). The narrow peaks are from CH_2Cl_2 (5.33 ppm), Et_2O (3.43 and 1.15 ppm), pump oil (1.28 and 0.88 ppm), residual proton impurities in the solvent (5.32 ppm), and TMS (0.00 ppm) which was added as an internal reference for the normalization of diffusion coefficients, following the procedure described by Stalke *et al.*[2] Processing parameters (TopSpin 4.3.0[1]): SI = TD, LB = 1.00 Hz.

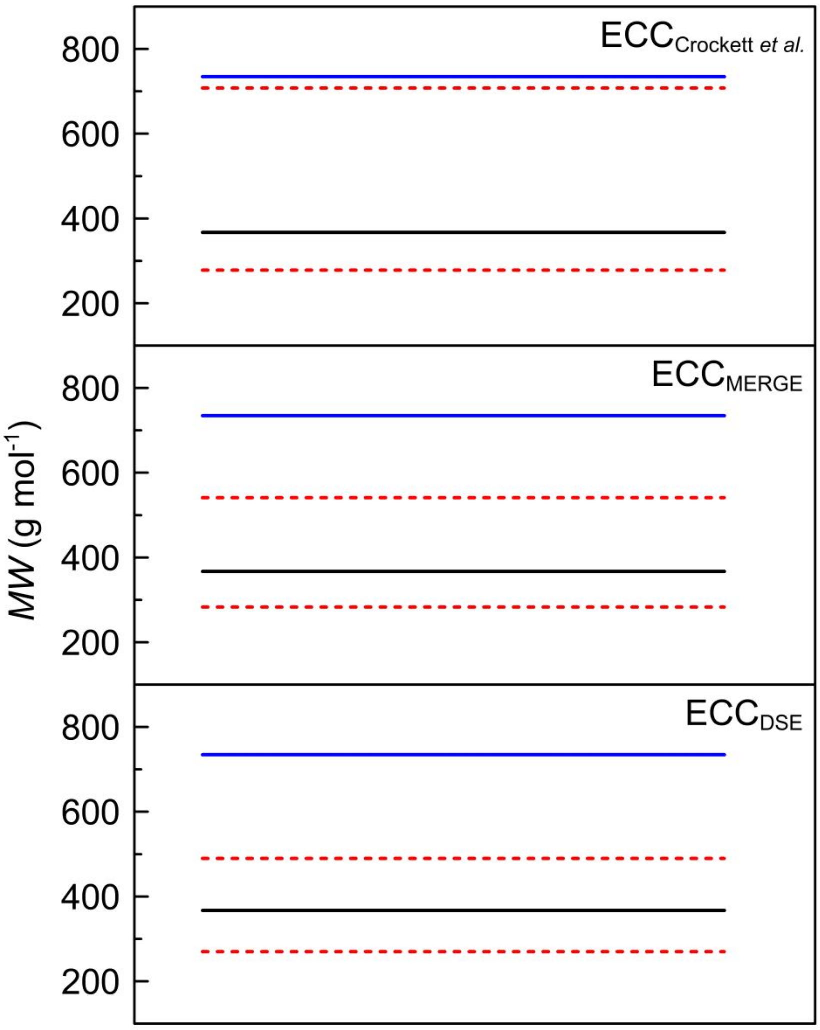

Figure S5. Graphical representation of estimated and calculated *MW*s. The dashed red lines represent the upper and lower limits of estimated *MW*s using each ECC. ECC_{DSE} and ECC_{MERGE} are from Ref.[2] while $ECC_{Crockett\ et\ al.}$ is the external calibration curve reported in Ref.[3]. The black and blue lines represent the calculated *MW*s of monomeric [VO(bdhb)] (**1'**) and dimeric **1**, respectively.

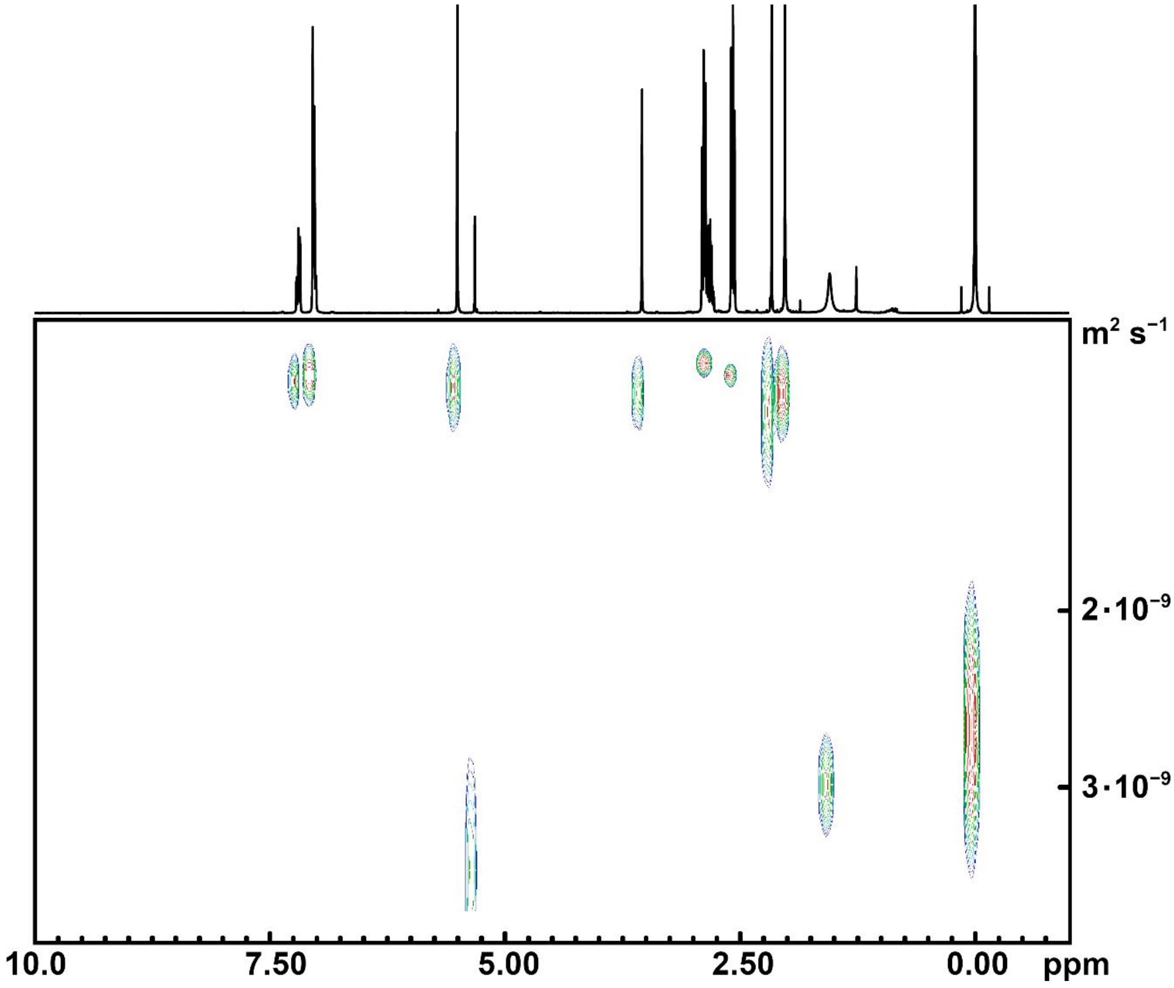

Figure S6. ^{1}H-DOSY spectrum of H_2bdhb in CD_2Cl_2 (400.13 MHz, 298 K). Impurity peaks are from H_2O (1.55 ppm), pump oil (1.26 and 0.89 ppm), and proton residuals in the solvent (5.32 ppm). TMS (0.00 ppm) was added as an internal reference for the normalization of diffusion coefficients, following the procedure described by Stalke *et al*.[2] Processing parameters (TopSpin 4.3.0[1]): SI = TD, LB = 0.30 Hz.

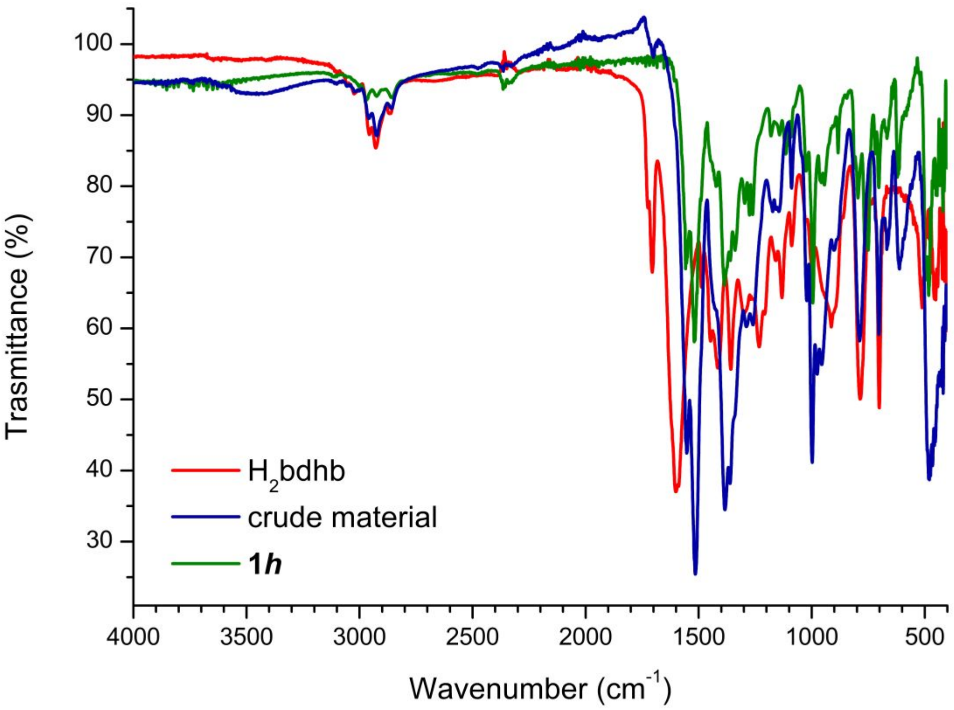

Figure S7. FT-IR spectra of H_2bdhb (red), the crude material (blue), and crystalline **1*h*** (green).

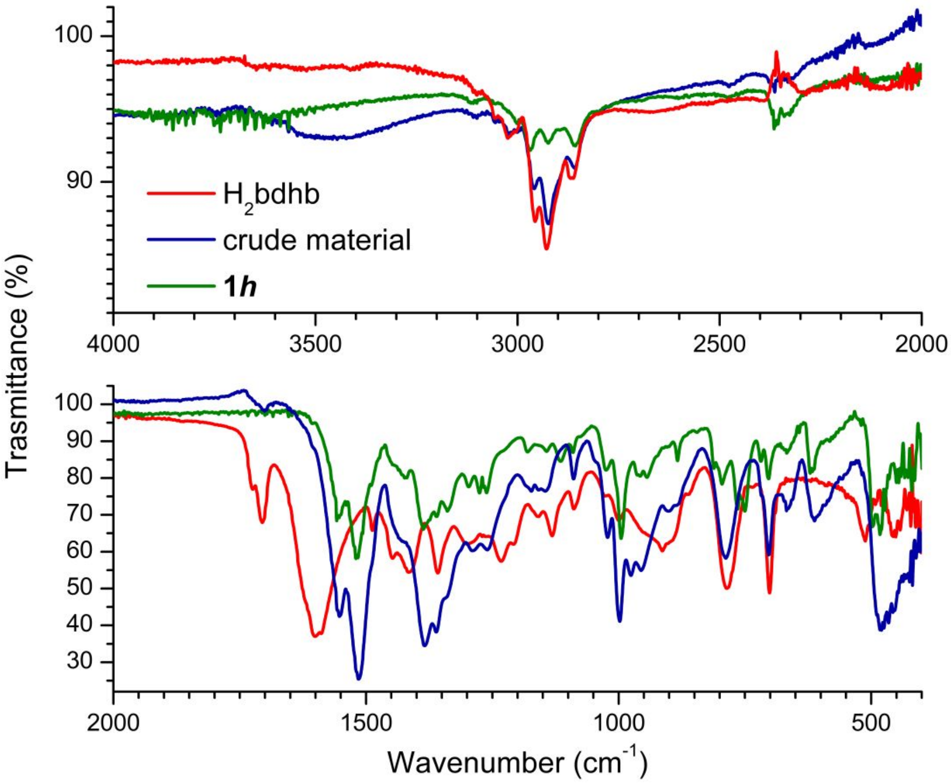

Figure S8. FT-IR spectra of H_2bdhb (red), the crude material (blue), and crystalline **1*h*** (green) in the region 4000–2000 cm^{-1} (top) and 2000–400 cm^{-1} (bottom). The presence of H_2O and EtOH molecules is reflected by the broad O-H stretching signal observed around 3450 cm^{-1}. The intense bands of the proligand between 1724 and 1602 cm^{-1}, which contain contributions from C=O and C=C stretching, are red shifted by approximately 100 cm^{-1} upon coordination.

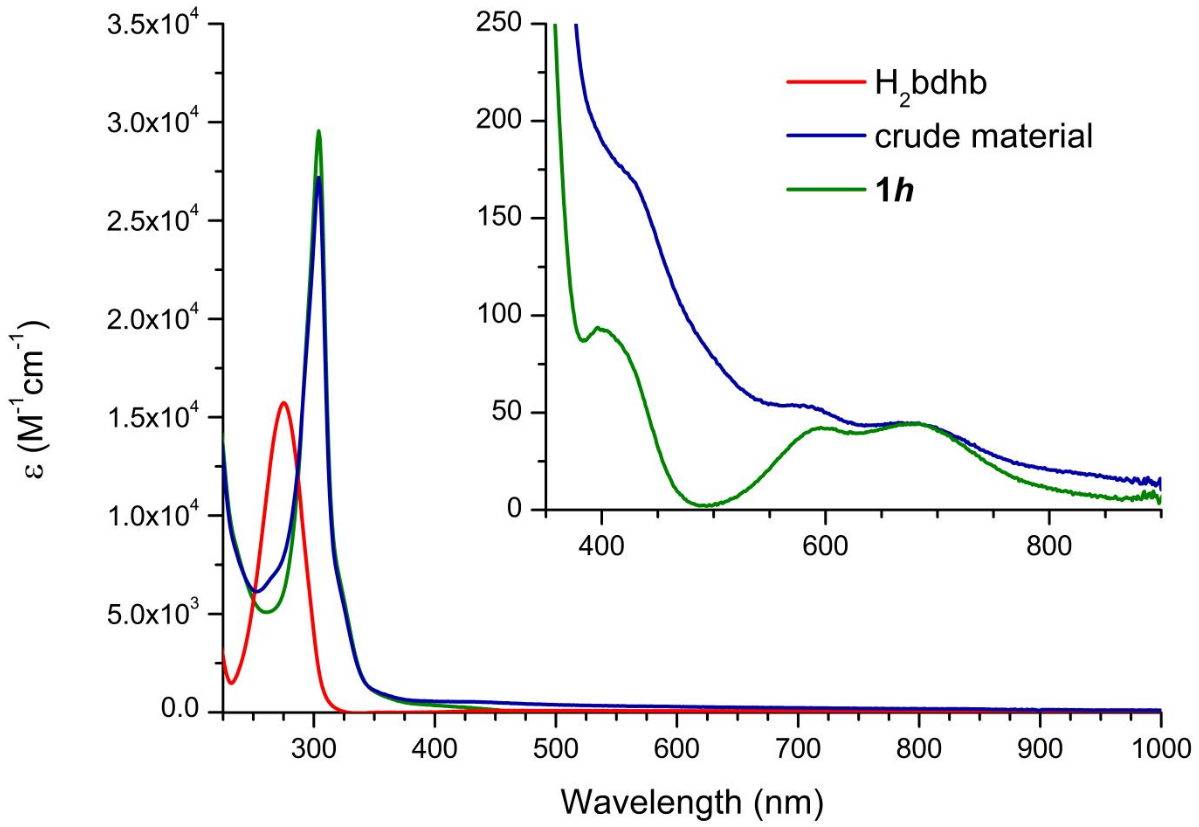

Figure S9. UV-Vis-NIR spectra of H_2bdhb (red), the crude material (blue), and crystalline **1*h*** (green) in CH_2Cl_2. The inset shows the characteristic absorption bands of vanadyl complexes.

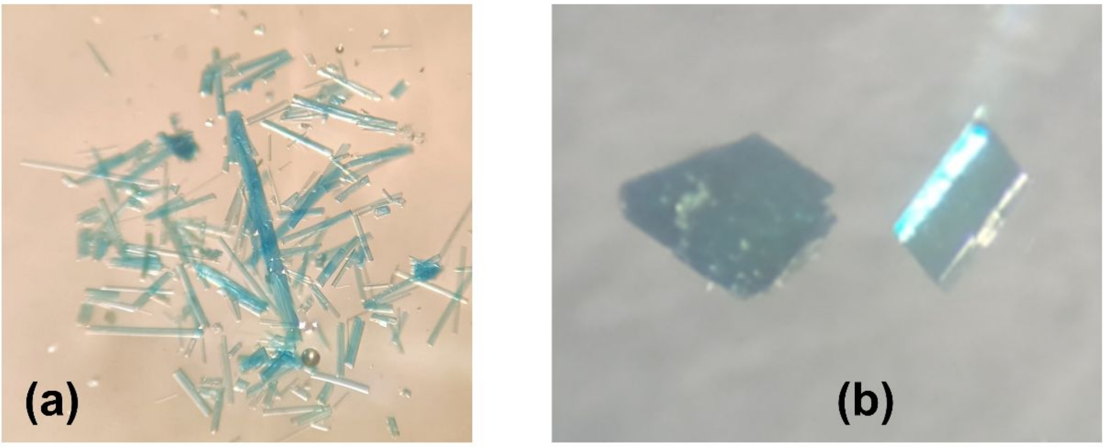

Figure S10. (a) Rod-like trigonal crystals of **1*h*** and (b) parallelogram-shaped crystals of monoclinic solvatomorph **1*m***.

Table S1. Crystal data and refinement parameters for **1*h***, **1*m***, and **1*a***.

Chemical formula	$C_{43.73}H_{59.26}Cl_{0.26}O_{11.90}V_2$ (**1*h***) [a]	$C_{39.69}H_{49.16}Cl_{0.21}O_{10.90}V_2$ (**1*m***) [b]	$C_{36}H_{40}O_{10}V_2$ (**1*a***)
Formula weight	886.42	809.81	734.56
T (K)	100(2)	100(2)	100(2)
Crystal size (mm^3)	0.900 × 0.172 × 0.172	0.120 × 0.060 × 0.040	0.250 × 0.150 × 0.050
Radiation	Cu-Kα (λ = 1.54178 Å)	Cu-Kα (λ = 1.54178 Å)	Cu-Kα (λ = 1.54178 Å)
Crystal system	trigonal	monoclinic	triclinic
Space group	$R\bar{3}$	$P2_1/c$	$P\bar{1}$
a (Å)	44.7168(10)	7.4835(2)	7.4876(4)
b (Å)	44.7168(10)	11.9824(3)	8.5547(5)
c (Å)	7.4408(3)	21.7146(5)	13.6688(8)
α (deg)	90	90	101.870(3)
β (deg)	90	96.492(2)	93.469(3)
γ (deg)	120	90	97.046(3)
V ($Å^3$)	12885.2(8)	1934.67(8)	847.03(8)
Z	9	2	1
ρ_{calcd} (g cm^{-3})	1.028	1.390	1.440
$\vartheta_{min}/\vartheta_{max}$ (deg)	3.424/72.372	4.098/68.373	3.317/68.471
Refls. collect./indep.	57672/5668	40710/3538	21316/3093
R_{int}	0.0540	0.0992	0.0598
Parameters/restraints	219/0	272/31	220/0
$R1/wR2$ (all data)	0.0330/0.0979	0.0498/0.0944	0.0533/0.1426
$R1/wR2$ ($I \geq 2\sigma(I)$)	0.0311/0.0966	0.0397/0.0898	0.0494/0.1384
GOF	1.098	1.064	1.124
ΔF max/min ($eÅ^{-3}$)	0.348/–0.338	0.323/–0.343	0.415/–0.776

[a]The given chemical formula and other crystal data include the solvent molecules in the channels, whose contribution to the scattering of X-rays was removed using the SQUEEZE routine.[4] [b]The given chemical formula corresponds to **1**·$0.895Et_2O$·$0.105CH_2Cl_2$ and results from structure refinement; ^{1}H-NMR and elemental analysis on the bulk material gave **1**·$0.80Et_2O$·$0.20CH_2Cl_2$.

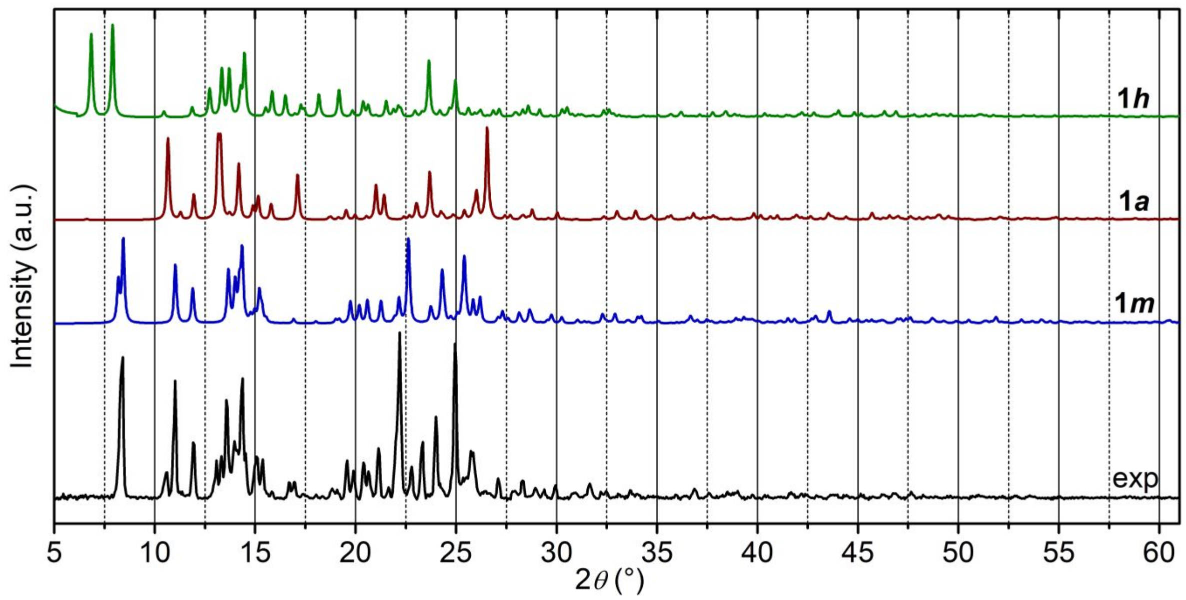

Figure S11. Background-subtracted PXRD pattern of the crystalline solid obtained upon transformation of **1*h*** (black), recorded at room temperature and with Cu-Kα radiation. The figure also shows the PXRD patterns of **1*m***, **1*a***, and **1*h*** simulated from their X-ray structures at 100 K using Mercury 2021.1.0,[5] with a full-width-at-half-maximum of 0.15° in 2θ.

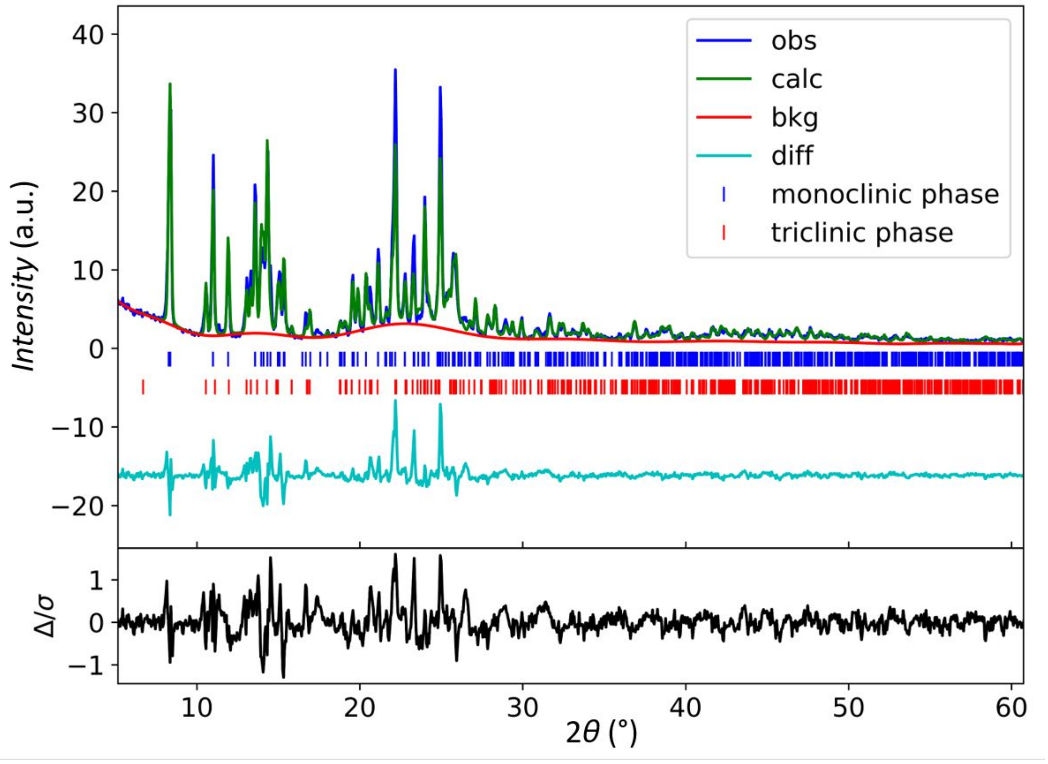

Figure S12. The Rietveld observed (blue line), calculated (green line) and difference curve (cyan line) of the refined pattern of the crystalline solid obtained upon transformation of **1*h***. The black curve at the bottom represents the weighted difference between the observed and calculated diffraction patterns. The ticks indicate the calculated positions of the reflections for the monoclinic (**1*m***) and triclinic (**1*a***) phases, with the color code indicated in the legend.

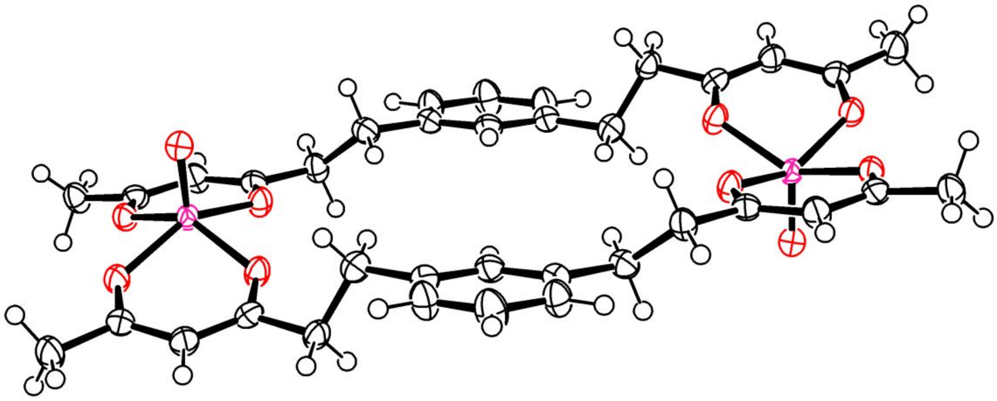

Figure S13. ORTEP-3[6] plot of the divanadium(IV) complex in **1*h***. Displacement ellipsoids are drawn at the 60% probability level while H atoms are depicted as spheres of arbitrary radius. Color code: pink (V), red (O), black (C and H).

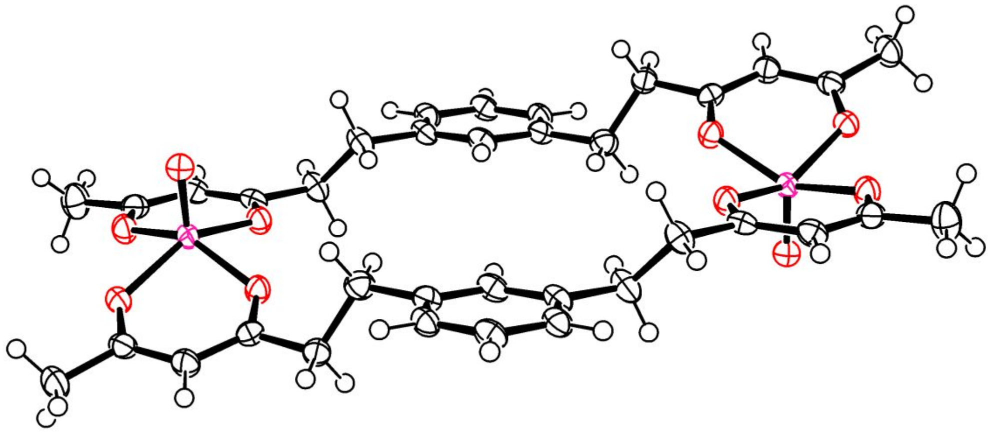

Figure S14. ORTEP-3[6] plot of the divanadium(IV) complex in **1*m***. Displacement ellipsoids are drawn at the 60% probability level while H atoms are depicted as spheres of arbitrary radius. The color code is the same as in Figure S13.

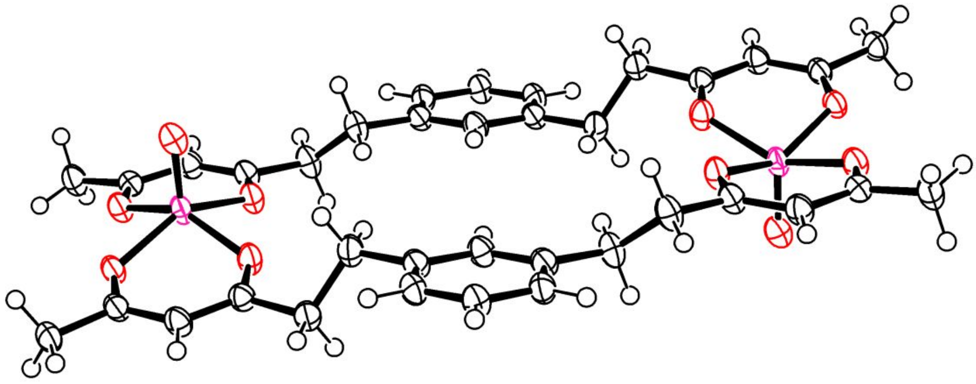

Figure S15. ORTEP-3[6] plot of the divanadium(IV) complex in **1*a***, omitting rotational disorder of CH_3 groups. Displacement ellipsoids are drawn at the 60% probability level while H atoms are depicted as spheres of arbitrary radius. The color code is the same as in Figure S13.

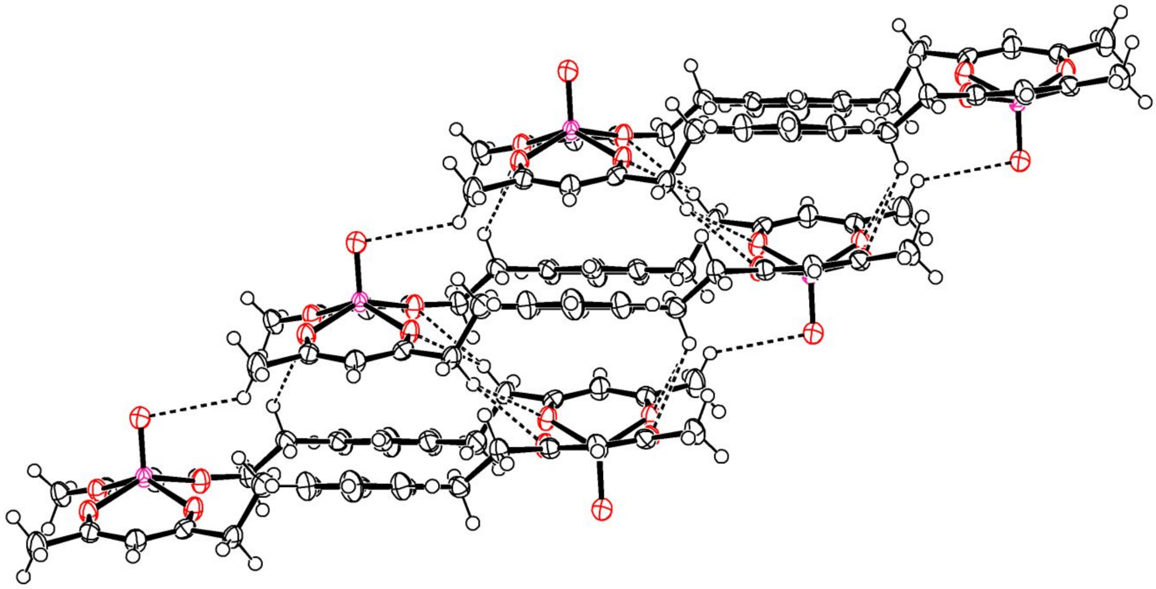

Figure S16. ORTEP-3[6] side view of divanadium(IV) complexes stacked into columns in **1*h***, with potential C–H···O hydrogen bonds depicted as dashed lines. Molecules are related by unitary translations along *z*. Displacement ellipsoids are drawn at the 60% probability level while H atoms are depicted as spheres of arbitrary radius. The color code is the same as in Figure S13.

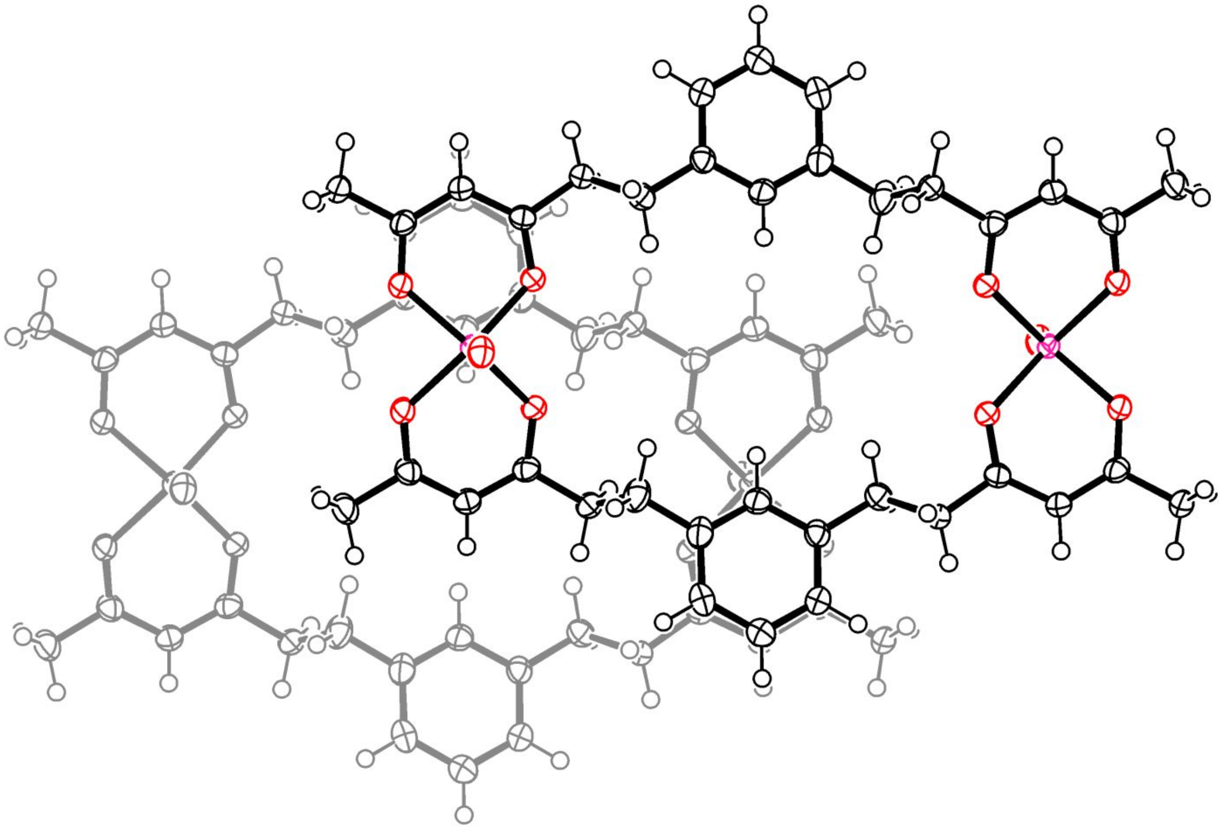

Figure S17. ORTEP-3[6] top view of two stacked divanadium(IV) complexes in **1*h***. Displacement ellipsoids are drawn at the 60% probability level while H atoms are depicted as spheres of arbitrary radius. The color code for the top molecule is the same as in Figure S13. The bottom molecule is translated by –1 along *z* and is depicted in grey.

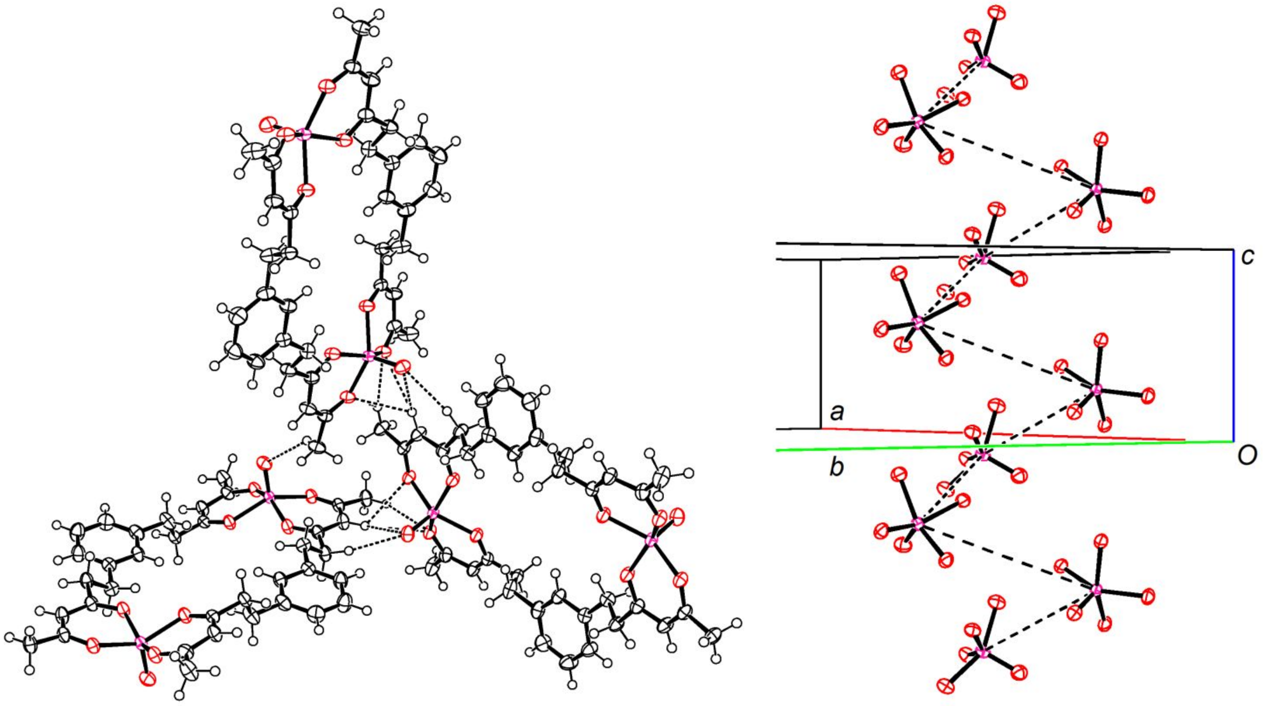

Figure S18. (Left) ORTEP-3[6] plot of three divanadium(IV) complexes related by a 3_1 axis in **1*h***, as viewed along *z* and with potential C–H···O hydrogen bonds depicted as dashed lines. (Right) The 1D helical chain of intermolecular dipolar interactions between metal centers spiralizing along a 3_1 axis in **1*h***, as viewed approximately orthogonal to *z* and including only the donor O atoms. The dashed lines highlight the shortest V···V distances in the crystal [7.1488(4) Å]. Displacement ellipsoids are drawn at the 60% probability level while H atoms are depicted as spheres of arbitrary radius. The color code is the same as in Figure S13.

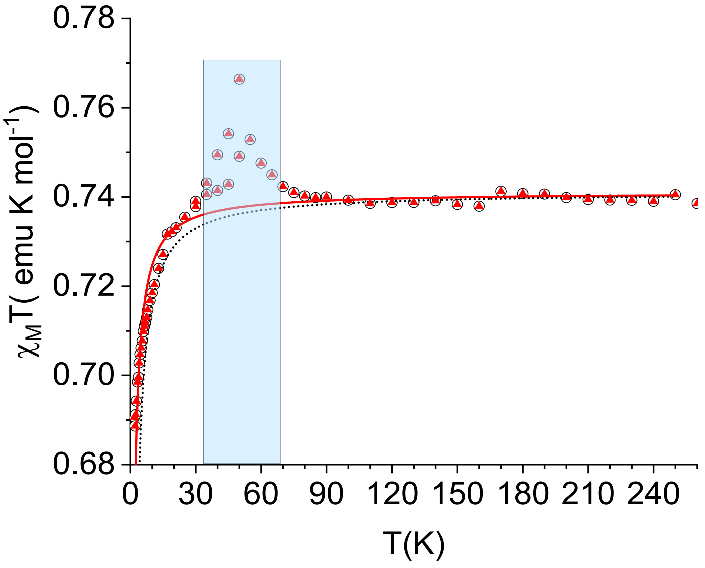

Figure S19. $\chi_M T$ vs. T curve recorded with an applied dc field of 1 kOe up to 30 K and of 10 kOe above 30 K on a microcrystalline sample of **1*h***, and best-fit curves obtained by using Eggert-Affleck-Takahashi equation for an antiferromagnetic regular spin-chain (red solid line) or Bleaney-Bowers equation for a dimer (black dashed line); see main text for more details. The feature at ca. 50 K is due to the onset of the antiferromagnetic transition of residual dioxygen present in the sample chamber. The data in the shaded area were not included in the fitting procedure.

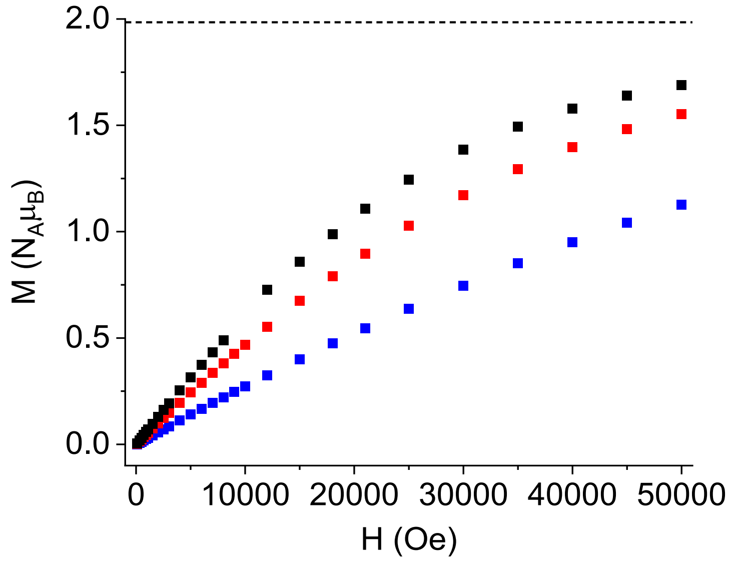

Figure S20. Field-dependent isothermal magnetization curves recorded at 1.9 K (black), 2.4 K (red), and 4.5 K (blue) on a microcrystalline sample of **1*h***. The horizontal dashed line is the saturation value expected for two uncorrelated S = 1/2 spins with g = 1.988.

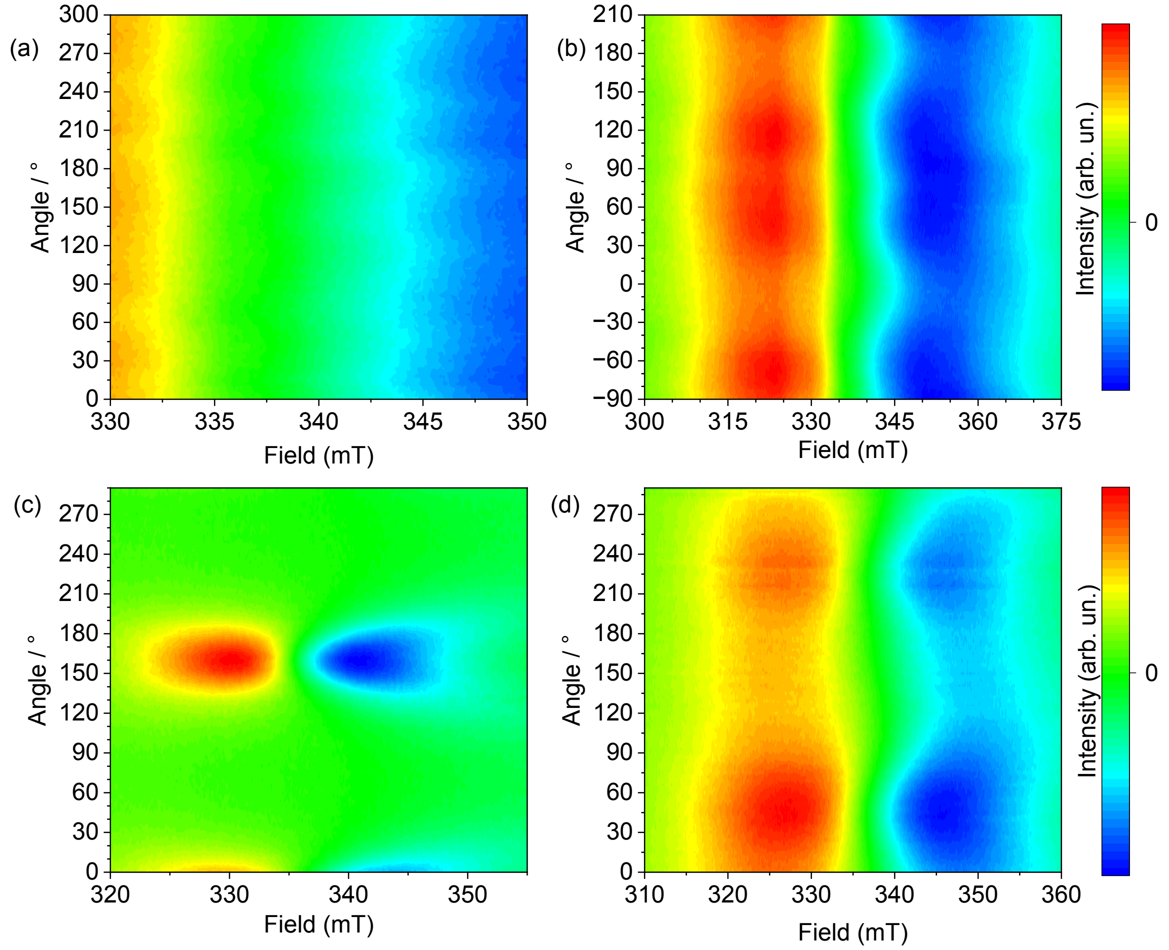

Figure S21. Upper panels: angle-dependent EPR spectra of **1*h*** at 50 K as the magnetic field is rotated in the *ab* plane (a) and from the *c* axis (angle = 0°) to the *ab* plane (b). Lower panels: angle-dependent EPR spectra of **1*m*** at 50 K as the magnetic field is rotated in the *ac* plane (c) and in a plane containing the *b* axis (d).

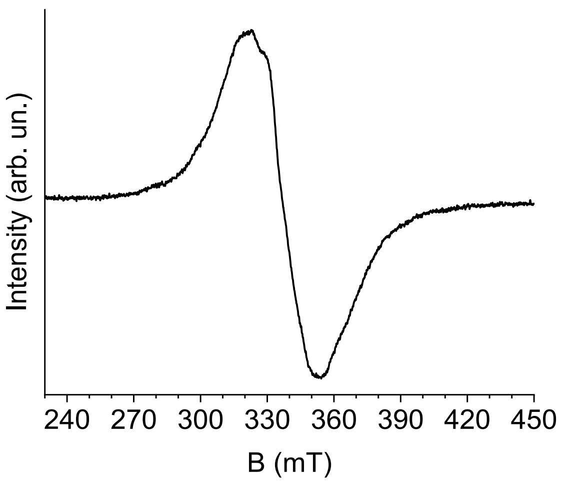

Figure S22. X-band CW-EPR spectrum of a single crystal of **1*h***, measured at 50 K with the magnetic field along the *c* axis.

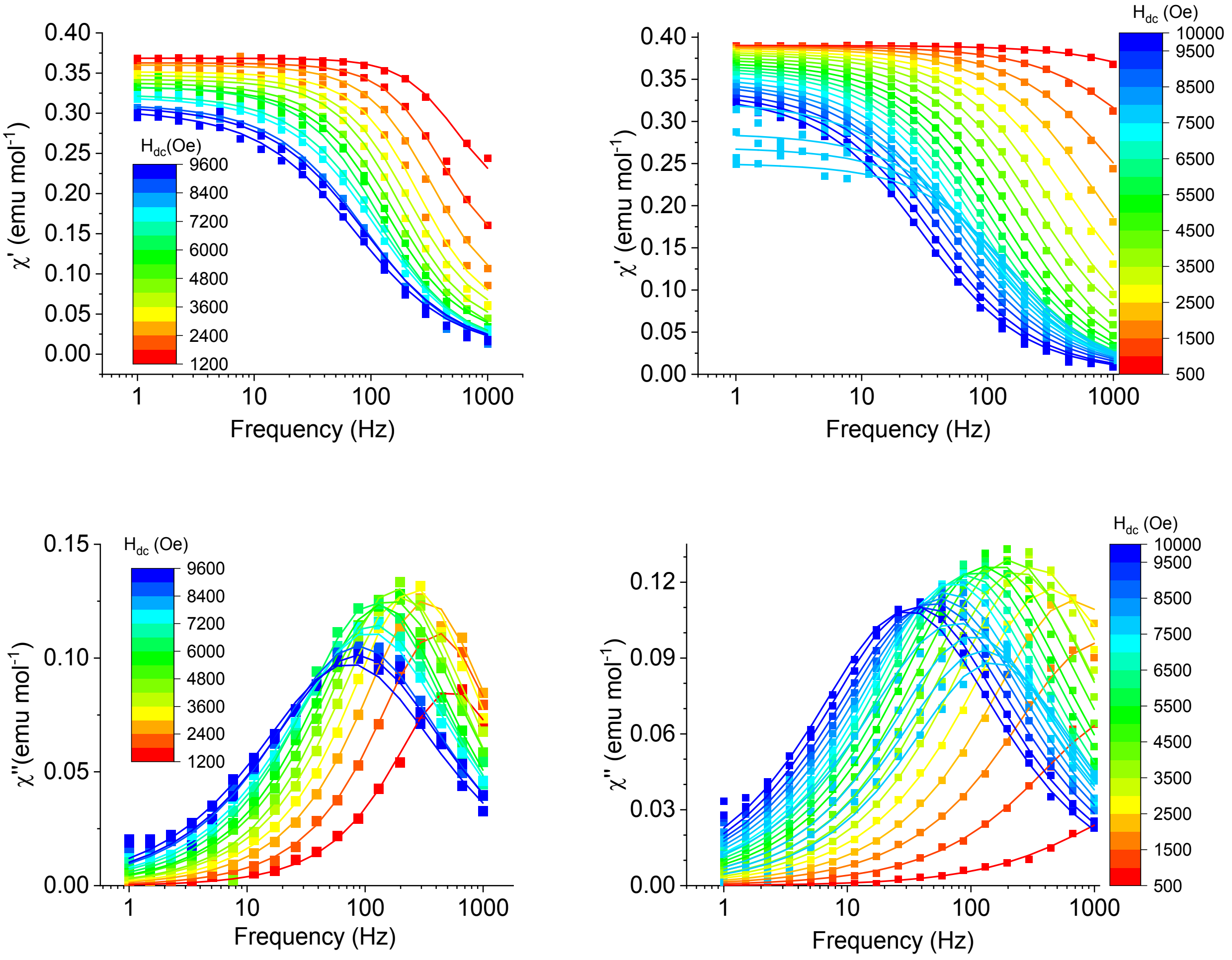

Figure S23. Variable-frequency ac susceptibility of **1*h*** (left) and **1*m*** (right) as a function of applied dc field, measured at 1.9 K. The solid lines represent the best-fit curves obtained using a generalized Debye model and the parameters reported in Tables S2 and S3.

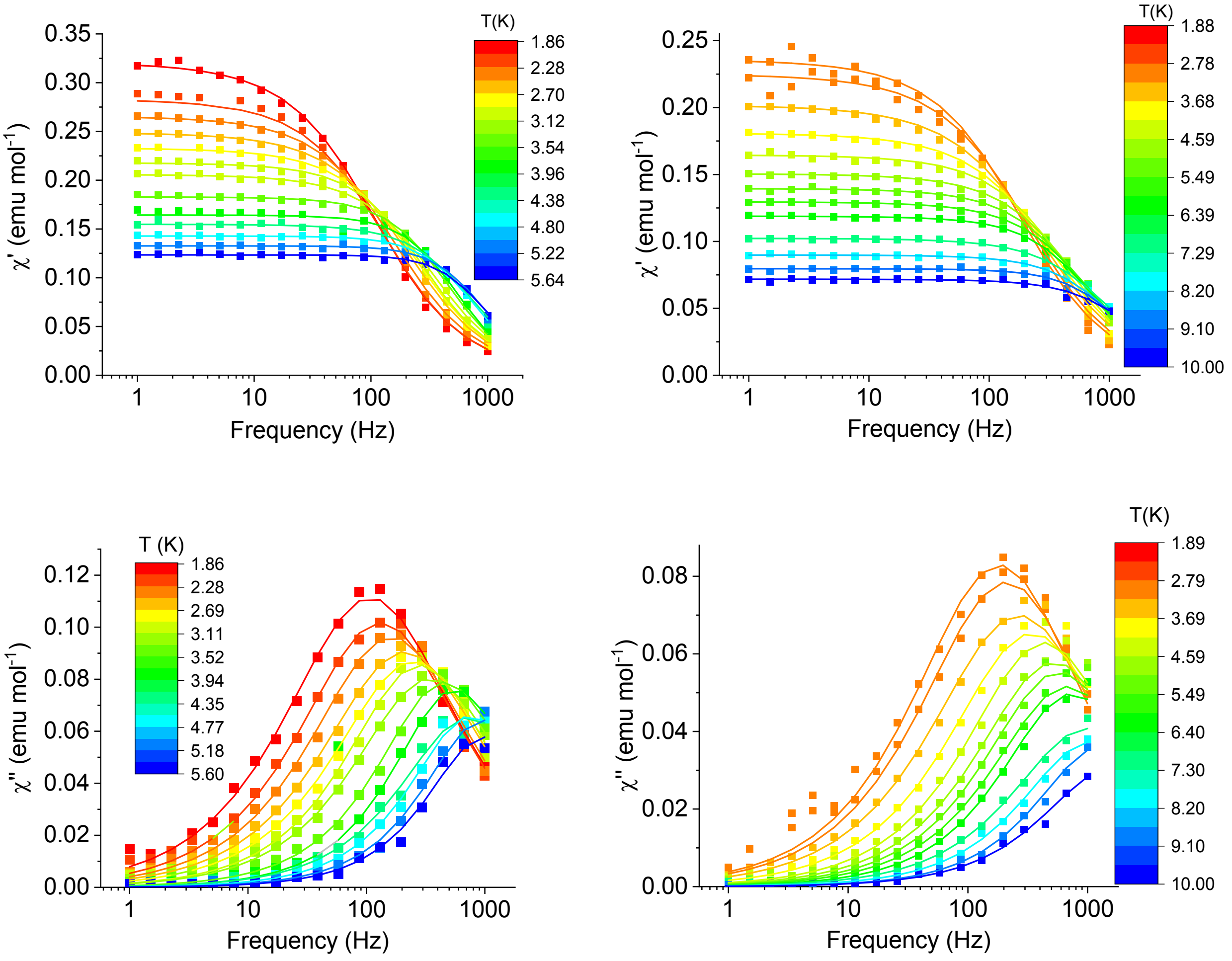

Figure S24. Variable-frequency ac susceptibility of **1*h*** (left) and **1*m*** (right) as a function of temperature, measured in dc fields of 7200 Oe and 7500 Oe, respectively. The solid lines are the best-fit curves obtained using a generalized Debye model and the parameters reported in Tables S2 and S3.

Table S2. Best-fit parameters for frequency dependent ac susceptibility curves of **1*h***.[a]

$\chi_T - \chi_S$ (emu mol^{-1})		τ (s)		α		χ_S (emu mol^{-1})		T (K)	H_{dc} (Oe)
0.1808	0.04914	2.970E-4	5.432E-5	0.03327	0.1514	0.1877	0.06398	1.892	1200
0.2474	0.02475	3.920E-4	4.838E-5	0.06864	0.04605	0.1154	0.02872	1.900	1800
0.2951	0.02292	4.850E-4	3.585E-5	0.1038	0.03910	0.06515	0.02827	1.895	2400
0.3090	0.02424	6.059E-4	4.504E-5	0.1077	0.03803	0.04261	0.03016	1.933	3000
0.3104	0.03039	7.554E-4	5.879E-5	0.1264	0.04060	0.03698	0.03943	1.899	3600
0.3147	0.05650	8.839E-4	9.398E-5	0.1177	0.07577	0.02772	0.07476	2.006	4200
0.3265	0.03606	9.750E-4	8.275E-5	0.1636	0.04327	0.01178	0.04588	1.900	4800
0.3159	0.07284	0.001165	1.083E-4	0.1544	0.08652	0.01710	0.09806	1.897	5400
0.3261	0.07987	0.001326	1.357E-4	0.1697	0.09056	0.007777	0.1068	1.894	6000
0.3223	0.07293	0.001367	1.807E-4	0.2125	0.07915	0.001650	0.09418	1.900	6600
0.3210	0.09123	0.001470	2.079E-4	0.2278	0.08880	3.726E-14	0.1211	1.897	7200
0.3123	0.08185	0.001810	2.394E-4	0.2438	0.08839	2.220E-14	0.1054	1.900	8400
0.3099	0.09961	0.001846	3.165E-4	0.2623	0.1036	2.739E-14	0.1270	1.933	9000
0.3053	0.1337	0.002182	5.120E-4	0.2763	0.1269	2.222E-14	0.1734	1.894	9600
0.2817	0.1633	0.001204	2.938E-4	0.2025	0.2011	0.001496	0.2154	2.164	7200
0.2625	0.06352	9.786E-4	9.675E-5	0.1936	0.08466	0.003157	0.08409	2.300	7200
0.2488	0.04207	7.552E-4	5.392E-5	0.1983	0.05920	3.395E-12	0.05540	2.500	7200
0.2331	0.03994	6.353E-4	7.076E-5	0.1822	0.06664	2.221E-14	0.05153	2.723	7200
0.2131	0.04598	5.637E-4	5.523E-5	0.1389	0.08545	0.004755	0.06154	2.900	7200
0.2059	0.03141	4.629E-4	3.590E-5	0.1558	0.06077	4.222E-13	0.04130	3.100	7200
0.1811	0.02784	3.459E-4	3.099E-5	0.08616	0.07040	0.001746	0.03673	3.499	7200
0.1643	0.07247	2.826E-4	9.446E-5	0.04642	0.2271	2.933E-14	0.08997	4.093	7200
0.1543	0.05028	2.171E-4	8.794E-5	0.1055	0.1315	5.686E-4	0.05661	4.299	7200
0.1404	0.02368	2.067E-4	2.893E-5	0.03916	0.08413	0.002372	0.02917	4.699	7200
0.1327	0.03480	1.658E-4	6.004E-5	0.02041	0.1105	1.182E-8	0.03667	5.099	7200
0.1155	0.04336	1.628E-4	8.246E-5	9.839E-13	0.1490	0.007865	0.04579	5.499	7200

[a]For each best-fit parameter ($\chi_T - \chi_S$, τ, α, χ_S) the second column gives the associated uncertainty.

Table S3. Best-fit parameters for frequency dependent ac susceptibility curves of **1*m***.[a]

$\chi_T - \chi_S$ (emu mol^{-1})		τ (s)		α		χ_S (emu mol^{-1})		T (K)	H_{dc} (Oe)
0.09361	0.1058	4.745E-5	9.248E-5	0.2382	0.2554	0.2965	0.1088	1.900	500
0.2028	0.05294	9.293E-5	4.223E-5	0.2564	0.07774	0.1867	0.05601	1.900	1000
0.2874	0.05066	1.479E-4	4.096E-5	0.2507	0.05174	0.1013	0.05418	1.900	1500
0.3300	0.02485	2.355E-4	2.497E-5	0.2362	0.02657	0.05712	0.02779	1.900	2000
0.3426	0.04368	3.549E-4	5.621E-5	0.2353	0.04734	0.04229	0.05018	1.900	2500
0.3612	0.03656	4.863E-4	4.926E-5	0.2237	0.03864	0.02142	0.04427	1.900	3000
0.3555	0.04209	6.798E-4	5.605E-5	0.2220	0.04386	0.02497	0.05416	1.900	3500
0.3773	0.06957	7.764E-4	1.054E-4	0.2368	0.06300	1.257E-13	0.08778	1.900	4000
0.3746	0.08261	9.925E-4	1.455E-4	0.2418	0.07331	2.235E-14	0.1056	1.900	4500
0.3694	0.09430	0.001256	2.117E-4	0.2357	0.08673	0.001598	0.1193	1.900	5000
0.3683	0.09228	0.001460	2.028E-4	0.2433	0.07785	2.312E-14	0.1195	1.900	5500
0.3655	0.1096	0.001719	2.883E-4	0.2451	0.09040	2.222E-14	0.1438	1.900	6000
0.3629	0.1068	0.001958	3.046E-4	0.2505	0.08976	2.221E-14	0.1400	1.900	6500
0.3577	0.1057	0.002221	3.825E-4	0.2474	0.09308	2.221E-14	0.1376	1.900	7000
0.3536	0.1131	0.002489	4.093E-4	0.2599	0.09175	2.221E-14	0.1491	1.900	7500
0.3496	0.08819	0.002828	3.847E-4	0.2655	0.07671	2.221E-14	0.1149	1.900	8000
0.3470	0.09692	0.003234	4.859E-4	0.2699	0.08225	2.352E-14	0.1266	1.900	8500
0.3408	0.09290	0.003714	4.777E-4	0.2681	0.07529	2.221E-14	0.1234	1.900	9000
0.3363	0.06633	0.004427	4.765E-4	0.2602	0.06043	2.222E-14	0.08641	1.900	9500

0.3326	0.06133	0.005193	5.884E-4	0.2622	0.05862	2.221E-14	0.07886	1.900	10000
0.3248	0.1274	0.002104	4.312E-4	0.2747	0.1164	2.354E-14	0.1651	2.100	7500
0.2862	0.08409	0.001501	1.956E-4	0.2297	0.09860	2.221E-14	0.1079	2.300	7500
0.2694	0.1106	0.001255	2.221E-4	0.2297	0.1254	3.053E-13	0.1483	2.500	7500
0.2505	0.1432	0.001050	2.346E-4	0.2168	0.1642	2.221E-14	0.1976	2.700	7500
0.2359	0.1059	8.718E-4	1.539E-4	0.2197	0.1295	2.221E-14	0.1458	2.900	7500
0.2250	0.06971	7.585E-4	1.008E-4	0.2241	0.09325	5.779E-14	0.09418	3.100	7500
0.2018	0.04489	5.989E-4	1.137E-4	0.2278	0.08128	2.221E-14	0.05554	3.499	7500
0.1809	0.02656	4.625E-4	5.797E-5	0.2034	0.05670	2.221E-14	0.03318	3.900	7500
0.1645	0.03230	3.664E-4	4.603E-5	0.1685	0.07553	2.228E-14	0.04296	4.299	7500
0.1505	0.02166	3.035E-4	3.931E-5	0.1647	0.06818	2.221E-14	0.02637	4.699	7500
0.1395	0.02185	2.738E-4	5.596E-5	0.1453	0.06865	2.241E-14	0.02463	5.099	7500
0.1295	0.01416	2.357E-4	3.120E-5	0.1434	0.05009	2.312E-14	0.01630	5.499	7500
0.1188	0.01493	2.154E-4	3.807E-5	0.1121	0.05255	5.329E-10	0.01651	5.999	7500
0.1023	0.01852	1.611E-4	3.799E-5	0.1430	0.07830	2.220E-14	0.02063	6.999	7500

[a]For each best-fit parameter (χ_T–χ_S, τ, α, χ_S), the second column gives the associated uncertainty.

Supplementary Note 1: EPR experiments on [VO(acac)$_2$]. The τ_r value for [VO(acac)$_2$] was derived by measuring a 3 mM solution of [VO(acac)$_2$] in toluene-d_8/CD_2Cl_2 (1:1 *v*/*v*) under the same experimental conditions used for **1*m*** (see Figure 6), yielding τ_r = 2.26×10^{-11} s and a hydrodynamic radius of 0.36 nm. Simulation analysis of the spectra estimated for τ_r a lower limit of 1.90×10^{-11} s and an upper limit of 2.66×10^{-11} s, which in turn correspond to a hydrodynamic radius of 0.34 and 0.38 nm, respectively.

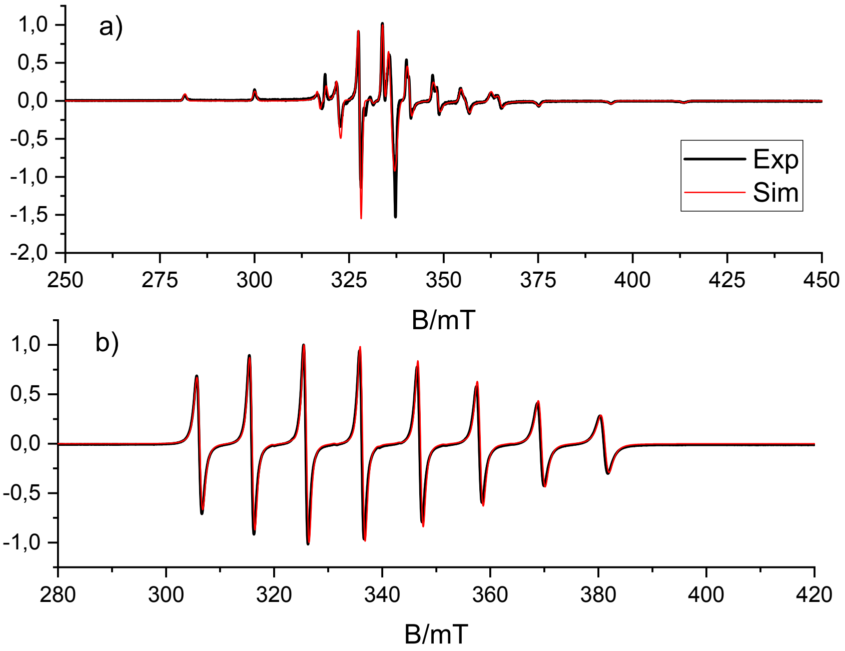

Figure S25. Experimental (black) and simulated (red) X-band CW-EPR spectra of [VO(acac)$_2$] recorded at 77 K (a) and RT (b) in toluene-d_8/CD_2Cl_2 (1:1 *v*/*v*).

Table S4. T_1 and T_m fitting parameters.

T (K)	T_1				T_m			
	t_fast (μs)	a_fast	t_slow (μs)	a_slow	t_fast (ns)	a_fast	t_slow (ns)	a_slow
10	2264	1004	13680	1184	2446	5844	13416	7517
15	878	3816	4602	4406	2343	8251	11792	14212
20	375	1306	2049	1780	1948	5367	9435	11918
30	125	2850	570	5185	161	122809	6512	20755
50	39	10217	121	15858	148	11306	5787	28242
70	15	27662	37	48838	-	-	4266	178133
90	4.5	210568	13.7	981586	-	-	2958	354545

Table S5. Best-fit parameters in equation (5).

A_{dir} (Hz)	A_{loc} (kHz)	$\hbar\omega_{loc}$ (cm^{-1})
4.0	101	83.7

Table S6. Differences in enthalpy and Gibbs free energy between *syn* and *anti* conformers of **1'**, along with their partition functions, computed at T = 298.15 K and p = 1.00 atm, in the gas phase and in different solvents, *viz.* pure toluene, pure CH_2Cl_2 and a mixture of toluene and CH_2Cl_2 (1:1 *v/v*).

phase	ΔH (kJ mol^{-1})	ΔG (kJ mol^{-1})	q(*syn*)	q(*anti*)
gas	3.1	4.9	0.122	0.878
toluene	2.9	4.6	0.133	0.867
CH_2Cl_2	0.8	2.9	0.238	0.762
mixture	1.4	3.0	0.230	0.770

Table S7. Eigenvalues of the **g** and **A** tensors (in MHz) computed for the two conformers of **1'** in the gas phase and in different solvents, *viz.* pure toluene, pure CH_2Cl_2 and a mixture of toluene and CH_2Cl_2 (1:1 *v/v*), using two different functionals. The average values are also depicted in Italics.

functional	phase	conformer	$g(\text{tot})_x$	$g(\text{tot})_y$	$g(\text{tot})_z$	A_x	A_y	A_z
BP86	gas	*syn*	1.99087	1.98815	1.96758	-133.7	-153.1	-438.1
		anti	1.99091	1.98791	1.96750	-128.5	-151.9	-433.8
		average	*1.99090*	*1.98794*	*1.96751*	*-129.2*	*-152.0*	*-434.3*
	toluene	*syn*	1.99064	1.98752	1.96877	-125.5	-147.3	-430.8
		anti	1.99066	1.98728	1.96852	-121.1	-146.4	-427.3
		average	*1.99066*	*1.98731*	*1.96855*	*-121.7*	*-146.5*	*-427.7*
	CH_2Cl_2	*syn*	1.99025	1.98637	1.97037	-113.1	-139.8	-420.2
		anti	1.99023	1.98613	1.97003	-109.2	-138.3	-416.7
		average	*1.99024*	*1.98619*	*1.97011*	*-110.1*	*-138.7*	*-417.5*
	mixture	*syn*	1.99044	1.98693	1.96977	-118.1	-142.5	-424.5
		anti	1.99045	1.98672	1.96943	-114.3	-141.5	-421.3
		average	*1.99045*	*1.98677*	*1.96951*	*-115.2*	*-141.7*	*-422.0*
TPSSh	gas	*syn*	1.99067	1.98839	1.96894	-150.3	-167.4	-461.8
		anti	1.99068	1.98824	1.96877	-146.2	-166.8	-458.7
		average	*1.99068*	*1.98826*	*1.96879*	*-146.7*	*-166.9*	*-459.0*
	toluene	*syn*	1.99046	1.98787	1.96989	-140.8	-160.3	-453.4
		anti	1.99047	1.98771	1.96964	-137.4	-159.9	-450.8
		average	*1.99047*	*1.98773*	*1.96967*	*-137.9*	*-160.0*	*-451.1*
	CH_2Cl_2	*syn*	1.99012	1.98695	1.97118	-127.5	-151.4	-441.6
		anti	1.99010	1.98679	1.97090	-124.4	-150.4	-439.0
		average	*1.99010*	*1.98683*	*1.97097*	*-125.1*	*-150.7*	*-439.6*
	mixture	*syn*	1.99029	1.98739	1.97071	-132.6	-154.4	-446.1
		anti	1.99030	1.98724	1.97041	-129.6	-153.8	-443.8
		average	*1.99030*	*1.98728*	*1.97048*	*-130.3*	*-154.0*	*-444.3*

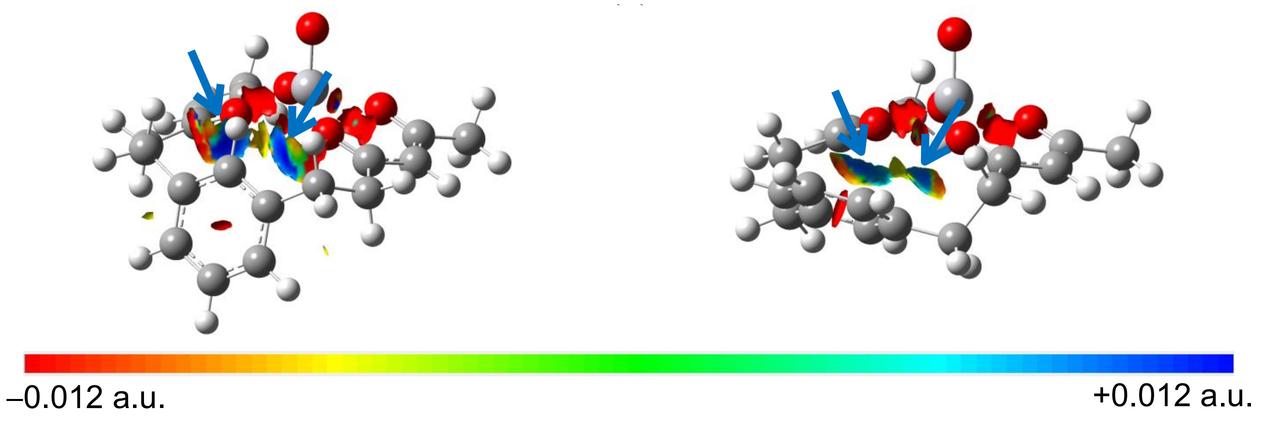

Figure S26. Intra-complex Non-Covalent Interactions (NCIs) in *anti*- (left) and *syn*-[(VO)(bdhb)] (right) conformers, depicted using the product of the sign of the second largest eigenvalue of electron density Hessian matrix and electron density, mapped on the reduced density gradient (RDG), with an isovalue $s = ½$. The blue arrows indicate attractive intra-complex NCIs established between the two β-diketonato O atoms closer to the phenyl ring and two C atoms of the phenyl ring (blue regions).

References

(1) *TopSpin 4.3.0*; Bruker AXS Inc.: Madison, WI, USA, 2023.

(2) Bachmann, S.; Neufeld, R.; Dzemski, M.; Stalke, D. New External Calibration Curves (ECCs) for the Estimation of Molecular Weights in Various Common NMR Solvents. *Chem. Eur. J.* **2016**, *22* (25), 8462–8465. https://doi.org/10.1002/chem.201601145.

(3) Crockett, M. P.; Zhang, H.; Thomas, C. M.; Byers, J. A. Adding Diffusion Ordered NMR Spectroscopy (DOSY) to the Arsenal for Characterizing Paramagnetic Complexes. *Chem. Commun.* **2019**, *55* (96), 14426–14429. https://doi.org/10.1039/C9CC08229H.

(4) Spek, A. L. PLATON SQUEEZE: A Tool for the Calculation of the Disordered Solvent Contribution to the Calculated Structure Factors. *Acta Crystallogr. Sect. C Struct. Chem.* **2015**, *71* (1), 9–18. https://doi.org/10.1107/S2053229614024929.

(5) Macrae, C. F.; Sovago, I.; Cottrell, S. J.; Galek, P. T. A.; McCabe, P.; Pidcock, E.; Platings, M.; Shields, G. P.; Stevens, J. S.; Towler, M.; Wood, P. A. Mercury 4.0 : From Visualization to Analysis, Design and Prediction. *J. Appl. Crystallogr.* **2020**, *53* (1), 226–235. https://doi.org/10.1107/S1600576719014092.

(6) Farrugia, L. J. WinGX and ORTEP for Windows: An Update. *J. Appl. Crystallogr.* **2012**, *45* (4), 849–854. https://doi.org/10.1107/S0021889812029111.
